# The Effects of a Meldonium Pre-Treatment on the Course of the LPS-Induced Sepsis in Rats

**DOI:** 10.3390/ijms23042395

**Published:** 2022-02-21

**Authors:** Siniša Đurašević, Aleksandra Ružičić, Iva Lakić, Tomislav Tosti, Saša Đurović, Sofija Glumac, Snežana Pejić, Ana Todorović, Dunja Drakulić, Sanja Stanković, Nebojša Jasnić, Jelena Đorđević, Zoran Todorović

**Affiliations:** 1 Faculty of Biology, University of Belgrade, 11000 Belgrade, Serbia; a.ruzicic@bio.bg.ac.rs (A.R.); djiva@bio.bg.ac.rs (I.L.); jasnicn@bio.bg.ac.rs (N.J.); jelenadj@bio.bg.ac.rs (J.Đ.); 2 Faculty of Chemistry, University of Belgrade, 11000 Belgrade, Serbia; tosti@chem.bg.ac.rs; 3 Institute of General and Physical Chemistry, University of Belgrade, 11000 Belgrade, Serbia; sasatfns@uns.ac.rs; 4 School of Medicine, University of Belgrade, 11000 Belgrade, Serbia; sofijaglumac09@gmail.com (S.G.); zoran.todorovic@med.bg.ac.rs (Z.T.); 5 Vinča Institute of Nuclear Sciences—National Institute of Serbia, University of Belgrade, 11000 Belgrade, Serbia; snezana@vin.bg.ac.rs (S.P.); anato@vin.bg.ac.rs (A.T.); drakulic@vin.bg.ac.rs (D.D.); 6 Centre for Medical Biochemistry, University Clinical Centre of Serbia, 11000 Belgrade, Serbia; sanjast2013@gmail.com; 7 Faculty of Medical Sciences, University of Kragujevac, 550601 Kragujevac, Serbia; 8 University Medical Centre “Bežanijska Kosa”, 11080 Belgrade, Serbia

**Keywords:** sepsis, liver, kidney, heart, inflammation, oxidative stress, lipidomics, rats

## Abstract

A dysregulated and overwhelming response to an infection accompanied by the exaggerated pro-inflammatory state and metabolism disturbance leads to the fatal outcome in sepsis. Previously we showed that meldonium, an anti-ischemic drug clinically used to treat myocardial and cerebral ischemia, strongly increases mortality in faecal-induced peritonitis (FIP) in rats. We postulated that the same mechanism that is responsible for the otherwise strong anti-inflammatory effects of meldonium could be the culprit of the increased mortality. In the present study, we applied the LPS-induced model of sepsis to explore the presence of any differences from and/or similarities to the FIP model. When it comes to energy production, despite some shared similarities, it is evident that LPS and FIP models of sepsis differ greatly. A different profile of sympathoadrenal activation may account for this observation, as it was lacking in the FIP model, whereas in the LPS model it was strong enough to overcome the effects of meldonium. Therefore, choosing the appropriate model of sepsis induction is of great importance, especially if energy homeostasis is the main focus of the study. Even when differences in the experimental design of the two models are acknowledged, the role of different patterns of energy production cannot be excluded. On that account, our results draw attention to the importance of uninterrupted energy production in sepsis but also call for much-needed revisions of the current recommendations for its treatment.

## 1. Introduction

Sepsis is a life-threatening condition, characterized by the exaggerated pro-inflammatory response and disturbed lipid metabolism leading to sequential organ failure [1,2]. Based on the data for 2005–2018, the ICU-, hospital- and 28/30-day mortality of patients from North America and Europe were 37.3%, 39.0% and 36.7%, respectively [3].

Previously we showed that meldonium, an anti-ischemic drug clinically used to treat myocardial and cerebral ischemia [4], strongly increases (50/100%) mortality in faecal-induced sepsis in rats [5]. Paradoxically, it appears that the same mechanism responsible for the otherwise strong anti-inflammatory effects of meldonium [6,7] could also be responsible for the increased mortality in sepsis. By inhibiting both the biosynthesis and transport of L-carnitine, meldonium prevents long-chain FFAs from entering mitochondria [8] and redirects them to peroxisomes instead, where they are metabolised into medium- and short-chain metabolites, some of which are further oxidised in mitochondria [9]. In this way, meldonium protects mitochondria from the accumulation of toxic long-chain FFA intermediates and reduces the risk of mitochondrial oxidative injury [10]. Despite its protective effects, by disturbing FFAs catabolism, meldonium impairs energy production, which is crucial in the prognostic assessment of sepsis [11]. Previously, it has been postulated that organ failure in sepsis is related to ischemia-induced tissue injury, but it became evident that, in sepsis, a normal amount of oxygen is delivered to the tissues [12,13]. Therefore, the decreased ability of tissues to use oxygen despite its availability suggests that a sort of metabolic ischemia is present in sepsis due to impaired energy homeostasis [14].

There are several experimental approaches for sepsis induction in animals, such as caecal ligation/puncture (CLP), colon ascendant stent plantation (CASP), faecal-induced peritonitis (FP) or lipopolysaccharide (LPS)-induced sepsis [15]. While the CLP, CASP and FIP are considered models of choice for mimicking human sepsis [16,17], the main conclusion of the International Expert Consensus for Pre-Clinical Sepsis Studies [18] was that the LPS model is not appropriate for replicating human sepsis [19]. Its main restriction is the fact that lipopolysaccharide represents a single component of the complex pathogen-associated molecular patterns released by gram-negative organisms, in contrast with the host–pathogen interactions of gram-positive organisms and polymicrobial sepsis in humans [20]. However, the LPS model is still the most common experimental toxaemia model [21], due to its high reproducibility and rendering of clinically significant conditions, such as meningococcaemia, bacteraemia and antibiotic-induced endotoxin release in septic shock [22].

Considering the above, we used the LPS model of sepsis to explore the presence of any differences and/or similarities to our previous model of sepsis induction [5]. Rats were pre-treated for four weeks with meldonium in 300 mg/kg b.m./day dosage and underwent sepsis induction by a single intraperitoneal injection of LPS (5 mg LPS/1 mL saline/1 kg b.m.).

To evaluate meldonium metabolic and lipidomics action, concentrations of L-carnitine, triglycerides (TGAs), FFAs, glycerol, glucose, lactic acid and inositol were measured in the liver, kidney and heart. Since meldonium upregulates expression of the peroxisome proliferator-activated receptor γ coactivator 1α (PGC-1α) [23], a transcriptional coactivator of the genes encoding proteins that participate in mitochondrial biogenesis and function [24], its expression level was also measured in the liver, kidney and heart of experimental animals. Adrenergic stimulation is of great importance for lipid metabolism efficiency, so adrenaline (ADR) and noradrenaline (NOR) concentrations were measured in both plasma and adrenal glands.

The extent of tissue injury was assessed by tissue histology and by measuring serum troponin T, creatinine and urea concentrations, as well as serum activity levels of alanine aminotransferase (ALT), aspartate aminotransferase (AST) and alkaline phosphatase (ALP). In all three tissues, the Bax/Bcl-2 ratio and level of high-mobility group box 1 protein (HMGB1) were measured as markers of apoptosis and necrosis, respectively. Since the HMGB1 expression is closely associated with the cytokine-mediated nuclear factor kappa B (NF-kB) signalling pathways in sepsis [25], we examined its activated phospho-NF-κB p65 form (p-NF-κB p65) in the liver, kidney and heart, as well as the serum level of tumour necrosis factor alpha (TNFα) [26].

Oxidative status was investigated by measuring the activity of copper-zinc superoxide dismutase (CuZnSOD), manganese superoxide dismutase (MnSOD), catalase (CAT) and glutathione peroxidase (GSH-Px) in the liver, kidney and heart, together with the tissue level of nuclear factor erythroid 2-related factor 2 (Nrf2), as one of the main regulators of both antioxidative and anti-inflammatory cell response [27]. The ratio of reduced and oxidised glutathione (GSH/GSSG) was measured as the marker of the tissue pro/antioxidant status. The level of malondialdehyde was assessed as a measure of the polyunsaturated fatty acids’ peroxidation (LPO).

## 2. Results and Discussion

**Figure 1:**
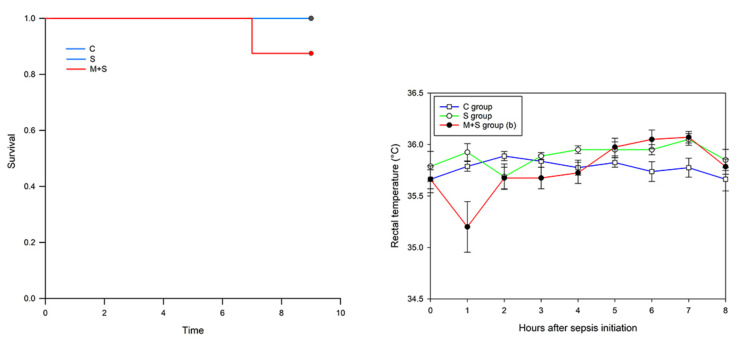
Survival rate analysis (**left**) and time-course curve of rectal temperature recordings (**right**) in rats of control (C), sepsis (S) and meldonium + sepsis (M + S) groups. Due to the overleaping, the C and S groups on the rectal temperature graph are marked with the same color (blue). The number of animals per experimental group: *n* = 8. The data on the rectal temperature recordings are given as timepoints mean ± standard error. Minimal significant level: *p* < 0.05. Significantly different: b with respect to the S group.

Survival rate and rectal temperature were monitored every hour for eight hours following the sepsis induction (Figure 1). Within this timeframe, death occurred in the M + S group only. Specifically, one animal in this group (12.5%, or 1/8 animals) died six hours after the LPS injection (Figure 1), although without statistical significance (Log-Rank significance *p* < 0.368). In comparison to the data from our previous model of sepsis induction [5], it can be concluded that mortality in both S and M + S groups was far higher in the FIP model. As opposed to the LPS, in the FIP model mortality rates were 12.5% and 50% in the S group and M + S group, respectively.

Rectal temperature, analyzed as the Area Under Curve (AUC) value, was significantly lower in the M + S group in comparison to the S group (AUC values 277.08 ± 8.71 vs. 287.22 ± 0.20, respectively, *p* < 0.007). This is in contrast to the FIP model, in which no differences were observed between the groups. When considering a decrease in body core temperature as a potent marker of sepsis severity [28], it is unclear why animal mortality was much lower in the LPS model of sepsis. The differences observed between the two models govern suspicion on the underlying mechanisms of sepsis induction and question the suitability of adhering to the same timeframe in both models (i.e., 8 h sepsis duration).

To examine the extent of organ injury, serum levels of AST, ALT, ALP, Troponin T, urea and creatinine were analysed (Table 1). AST, ALT and ALP are enzymes with a key role in the amino acid metabolism in a variety of organs including liver, brain, kidneys, pancreas, bile duct, gallbladder, spleen, heart and seminal vesicles [29]. If any of these tissues suffer damage, these enzymes are released into the bloodstream. The presented results show that the pattern of ALT activity among the groups was similar to the pattern observed in the FIP model [5], i.e., ALT was increased in the S group and decreased in the M + S group. Similarly, ALP activity also increased in the LPS sepsis, but no further increase was observed in the M + S group, as was the case in the FIP model. In addition, as opposed to the FIP model, in which AST activity level increased in the S group but was reduced by the meldonium pre-treatment, no such changes were observed in the LPS model. Since elevated ALT and ALP serum levels are markers of primarily hepatic injury, as they have the highest activity levels in the hepatocytes, these findings point to the liver as the primary site of the LPS-induced injury and demonstrate the hepatoprotective effects of meldonium.

Troponin T is a well-established marker of heart function as its serum concentration increases when damage to the heart muscle occurs. The presented results show that its concentration is increased by almost four-fold in the S group whereas meldonium pre-treatment ameliorates this increase by 50% (Table 1). The same pattern of changes in both models of sepsis denotes the cardioprotective effects of meldonium.

Serum urea and creatinine levels were measured as markers of kidney function. This analysis was not included in our previous experiment. As both urea and creatinine are end products of nitrogen metabolism filtered by the glomerulus, their serum concentrations are indirect measures of the glomerular filtration rate (GFR) [30]. Kidney disease, irrespective of its cause, is associated with decreased GFR, resulting in a rise in serum urea, and especially creatinine concentration [31]. Our results show that LPS sepsis induces a two-fold increase in urea serum concentration without changes in creatinine concentration (Table 1). While meldonium pre-treatment reduced LPS-induced urea concentration rise by almost 40%, it exerted no impact on the creatinine concentration (Table 1). The fact that creatinine concentration remained unchanged could be explained by the underlying mechanisms of its synthesis. Creatinine formation primarily occurs in the kidneys, whereas urea is mainly synthesised (99%) in the liver [32]. The rate of urea synthesis and, consequently, its serum levels increase in inflammation [33]. Therefore, increased serum urea accompanied by the absence of serum creatinine increase indicates primarily liver inflammation, as shown in the case of lipopolysaccharide administration [34].

**Figure 2:**
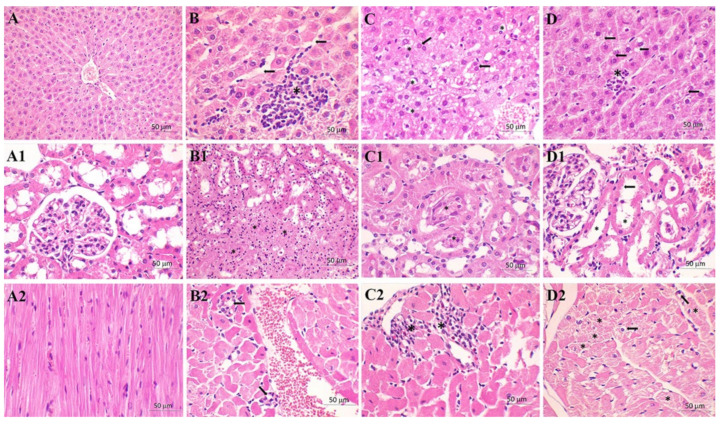
Tissue histology analysis (H&E stain, 400×). Liver: (**A**) control (C) group—normal histological structure of the hepatic tissue; (**B**) sepsis (S) group—larger zone of centrilobular necrosis (asterisk) with sinusoidal dilatation (arrow); (**C**) sepsis (S) group—hydropic degeneration (vacuolization of the hepatocytes cytoplasm, asterisk) and binucleation of hepatocytes (usually seen in regenerating cells, arrow); (**D**) meldoniuM + Sepsis (M + S) group—rare spotty necrosis (asterisk), several adjacent hepatocytes are absent and replaced by inflammatory cells and focal steatosis in the surrounding preserved hepatocytes (arrow). Kidney: (**A1**) control (C) group—normal histological structure of the renal tissue; (**B1**) sepsis (S) group—severe tubular necrosis with complete loss of parenchymal architecture (asterisk); (**C1**) sepsis (S) group—intratubular obstruction due to the denuded epithelium and cellular debris and bleb formation (asterisk); (**D1**) meldonium + sepsis (M + S) group—absence or thinning of brush-border of the proximal tubule (arrow) with dilated proximal tubule (asterisk) with flattening of tubular epithelium. Heart: (**A2**) control (C) group—normal histological structure of the heart tissue; (**B2**) sepsis (S) group—Focal inflammatory cell infiltration (arrow) in the myocardium, consisting of mononuclear cells, occasionally occurs. This change is sometimes accompanied by slight myocardial necrosis; (**C2**) meldonium + sepsis (M + S) group—diffuse interstitial mononuclear infiltration (asterisk) with greater loss of myocytes; (**D2**) meldonium + sepsis (M + S) group—intracellular vacuolization (arrow) and a loss of cross-striations (asterisk) in subendocardial zone of heart.

To further explore the extent of organ injury, we examined the liver, kidney and heart histology (Figure 2), with the histology analysis results presented by the score system (Table 2). The liver tissue injury was assessed according to the Suzuki grading scale, which includes five grades, based on the degree of the most common finding in the septic liver (i.e., tissue congestion, cell ballooning and necrosis area—all nonspecific) [35]. Since the most common cause of renal failure in patients with sepsis is acute tubular necrosis developing due to the microvascular alteration and pathologic inflammatory response [36], the hallmarks of kidney damage were tubular epithelial cell flattening (TF), brush border loss (BBL), tubular necrosis (TN) and tubular lumen obstruction (TO). To analyse heart samples, the Dallas criteria for the grading of the intensity and distribution of inflammatory infiltrate were used [37].

As seen in Table 2, regardless of the tissue, all the examined histological parameters were worsened in the S group (Table 2). Meldonium pre-treatment exerted a protective effect in the liver of animals from the M + S group, but further worsened the heart scores without altering kidney histological scores. A comparison between LPS and FIP models of sepsis revealed several differences. First, the extent by which LPS sepsis worsened the liver scores was twice as high in comparison to the FIP model [5], while the meldonium-mediated reduction in these changes was more prominent in the LPS sepsis than in the FIP sepsis. As opposed to the liver, kidney did not exhibit major differences between LPS- and FIP-induced sepsis. However, a contrast in the effects of meldonium was clear: in the LPS model, all scores in the meldonium pre-treated group were equal to or lower than the scores in the S group, in contrast to the FIP model, in which meldonium worsened all of the examined parameters.

When taken together, histology findings in the liver and kidney indicate that the LPS sepsis more severely affects the liver than the kidney. This is in line with our observation of liver damage supported by the rise in serum urea, AST and ALT in the S group (Table 1). Regarding the heart histology, it can be concluded that heart is the only organ in which meldonium caused deterioration regardless of the model of sepsis induction, with the extent of LPS-induced worsening being twice as large. However, this is in strong disagreement with the rise in serum Troponin T level in the S group and its decrease in the M + S group (Table 1).

**Figure 3:**
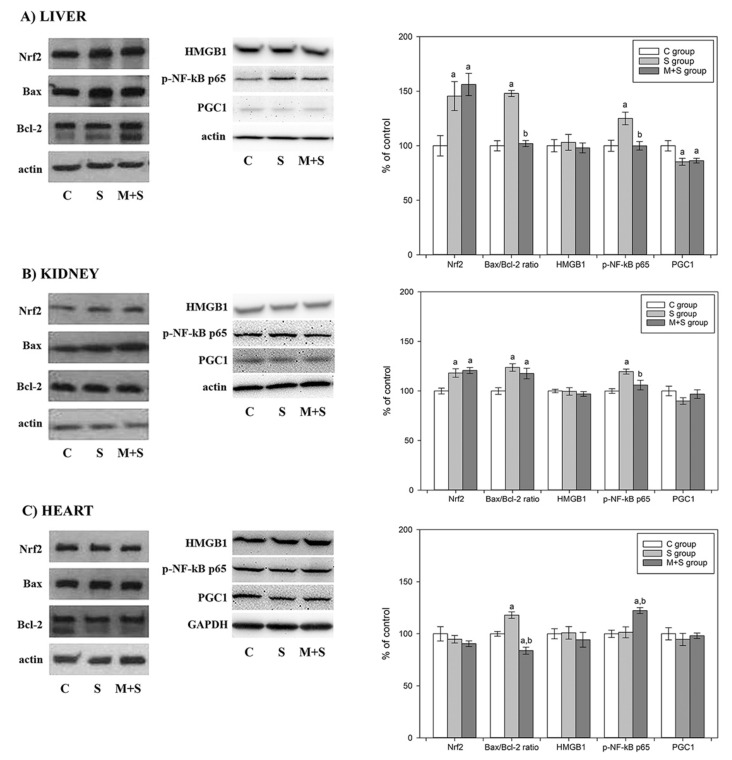
Representative immunoblots of Nrf2, Bax, Bcl-2, HMGB1, p-NF-κB p65, and PGC1 protein expression levels in (**A**) liver, (**B**) kidney and (**C**) heart in rats of control (C), sepsis (S) and meldonium + sepsis (M + S) groups. β actin was used as a loading control. Protein expression levels are calculated relative to actin and graphically presented as the percent of control (mean ± standard error). Minimal significant level: *p* < 0.05. Significantly different: ^a^ with respect to C; ^b^ with respect to S.

To clarify the histological findings, we examined the tissue inflammatory status (Figure 3). Namely, sepsis is characterized by the excessive release of inflammatory mediators, such as HMGB1 [38,39]. However, sepsis-induction by LPS had no effects on the tissue level of HMGB1, regardless of whether animals were pre-treated with meldonium or not (Figure 3A–C). This was an unexpected finding, in strong contrast to the changes observed in the FIP model (1.2-, 1.7- and 1.4-fold increase in the liver, kidney and heart, decreased by meldonium by 30, 34 and 18%, respectively) [5]. HMGB1 increase is considered a common marker of sepsis as it sustains a potentially injurious inflammatory response due to its role in recruiting, alerting and activating innate immune cells [39,40]. In addition, as a member of the Damage-Associated Molecular Pattern Molecules (DAMPs), HMGB1 serves as a necrotic marker since it is released by the stressed, damaged, or dying cells [41]. Altogether, in comparison to the FIP model, it can be concluded that the LPS-induced sepsis prompted much lower levels of tissue damage.

Since the proinflammatory action of HMGB1 includes the activation of NF-κB signalling pathways [25], we examined the changes in its phosphorylated form (p-NF-κB p65). LPS sepsis increased p-NF-κB p65 level by 25% in the liver and kidney (Figure 3A,B) but did not alter its cardiac level (Figure 3C). While meldonium reduced the liver and kidney p-NF-κB p65 level, returning it to the control level (Figure 3A,B), it unexpectedly increased it by 25% in heart (Figure 3C). This is another difference to the FIP model, in which sepsis increased the heart p-NF-κB p65 level, and meldonium successfully reduced these levels [5].

Upon encountering pathogen-associated patterns (PAMPs) or DAMPs [42], the host acute phase response initiates intracellular signal transduction pathways, leading to transcription and the release of pro-inflammatory cytokines, such as TNFα [43]. This is a pleiotropic cytokine, primarily synthesized by macrophages, dendritic cells and T lymphocytes [44], which can function either in its membrane-bound form, or be cleaved at the cell surface by a specific metalloproteinase [45]. Both membrane-bound and soluble TNFs bind to the transmembrane receptor, which then recruits various cytosolic proteins. A finely regulated cascade includes an unmasking of the p65 subunit of NF-κB, which then gains entry into the nucleus and promotes the transcription of various target genes [46].

As seen in Table 1, the serum level of TNFα increased in the S group and decreased in the M + S group, indicating an inflammatory increase in sepsis, and its reduction by meldonium. These results are consistent with the changes in p-NF-κB p65 level observed in the liver and kidney, since this also increased in the S group and decreased in the M + S group (Figure 3A,B). The changes in TNFα serum concentration may explain the worsening of the histological score present in all three organs (Table 2), as it is known that in the LPS-treated rats TNFα may be the predominant factor resulting in acute kidney [47] or cardiac injury [48]. Therefore, the increase in p-NF-κB p65 level in the heart of meldonium-treated animals (Figure 3C), accompanied by the worsened histological score (Table 2), remains unclear.

The NF-κB p65 signalling is involved in the regulation of cell apoptosis in different organs [49]. Apoptosis includes a delicate interplay of pro-apoptotic and anti-apoptotic members of the Bcl2 protein family, such as Bax and Bcl-2 proteins. While Bax promotes both apoptosis and necrosis, Bcl-2 blocks them [50]. Hence, the Bax/Bcl-2 ratio is considered an apoptotic marker, as it rises in the presence of pro-apoptotic events and lowers in the presence of anti-apoptotic events. As shown in Figure 3, the Bax/Bcl-2 ratio increased by 50% in the liver (Figure 3A) and by 25% in the kidney and heart of the S group (Figure 3B,C), indicating increased tissue apoptosis in LPS sepsis. In the M + S group, its ratio was decreased by 30% in the liver (Figure 3A) and by 25% in the heart (Figure 3C), suggesting the anti-apoptotic effects of meldonium. However, this effect was absent in kidneys of the same group (Figure 3B). When these results are compared to the results obtained in the FIP model, two major observations can be drawn. Firstly, the difference in the extent of the Bax/Bcl-2 rise in sepsis is evident. In the FIP model, sepsis induced a 3-, 2.5- and 1.4-fold increase of Bax/Bcl-2 ratio in the liver, kidney and heart, respectively [5]. Additionally, in the FIP model, meldonium did exhibit anti-apoptotic effects in kidneys, as demonstrated by a Bax/Bcl-2 ratio decrease. Overall, it seems that the LPS and FIP model of sepsis differ in their impact on the tissue apoptotic/necrotic relationship. Increasing HMGB1 and Bax/Bcl-2 FIP induces both apoptosis and necrosis, whereas the effects of LPS sepsis are limited to apoptosis, based on the unchanged HMGB1 levels. Finally, the cardiac changes in the Bax/Bcl-2 ratio may also explain the changes observed in the serum level of Troponin T (Table 1). Namely, cardiac Troponin T is released into serum as a result of myocardial injury [51], caused either by necrosis, or apoptosis [52]. Therefore, the increased serum Troponin T in the S group and its decrease in the M + S group (Table 1) are in correspondence with the increased cardiac Bax/Bcl-2 ratio in the S group, and its decrease in the M + S group (Figure 3).

We also investigated the protein expression of Nrf2, one of the most important inducible transcription factors that protects tissue, by activating the endogenous antioxidant system. Our results showed that LPS sepsis increased Nrf2 expression in the liver and kidney (Figure 3A,B), but not in the heart (Figure 3C). Meldonium pre-treatment had no additional impact on the Nrf2 expression (Figure 3A–C). Even though we did not include Nrf2 analysis in the FIP model, we previously showed that meldonium increases liver and the kidney Nrf2 expression level is otherwise decreased by the ischemia/reperfusion [6,7]. Therefore, it is unclear why sepsis increased Nrf2 expression and why meldonium exerted no effect. It may be that Nrf2 is a key factor in counteracting septic inflammation [53]. For instance, mortality in response to endotoxin-, caecal ligation and puncture-induced septic shock is much higher in Nrf2-deficient mice [54]. In addition, the Nrf2 increase protects from the LPS-induced deregulation of the innate immune response [55]. Hence, the Nrf2 increase that we observed in the LPS sepsis may represent a part of the cell’s survival strategy, which is effective enough irrespective of the meldonium pre-treatment.

PGC1α plays an important role in the diet-induced metabolic reprogramming in a variety of tissues, acting as an upstream regulator of lipid and glucose metabolism [56]. It has been suggested that meldonium could be involved in the PGC1α upregulation, by acting as an inhibitor of carnitine-palmitoyltransferase-1 (CPT-1), thus leading to the accumulation of acyl-CoA and fatty acids in the cytosol [57]. Our results showed that sepsis only significantly decreases PGC1α expression in the liver (Figure 3A). This result, which was not observed in the FIP model, is in line with novel studies showing a reduction in PGC1α content in the septic liver [58]. Recently, Di Cristo et al. demonstrated the meldonium-induced increase in PGC1α expression in a *Drosophila* model of Huntington’s disease [59]. However, meldonium did not induce changes in the PGC1α content in the examined organs in either of our septic models (Figure 3A–C). This discrepancy may be simply explained by the usage of different experimental animals.

Increased reactive oxygen species (ROS) production is a well-recognized prognostic marker of the sepsis outcome [60]. To date, several antioxidative enzymes have been shown to be upregulated by p-NF-κB 65 or Nrf2. One of the well-known NF-κB targets is MnSOD [61,62], while proposed targets also include CuZnSOD [63], CAT [64] and GSH-Px [65]. Once activated in the cytosol, Nrf2 trans-activates antioxidant response elements [66] that are present in a myriad of genes, including those coding for the CuZnSOD, GSH-Px and CAT [67].

**Figure 4:**
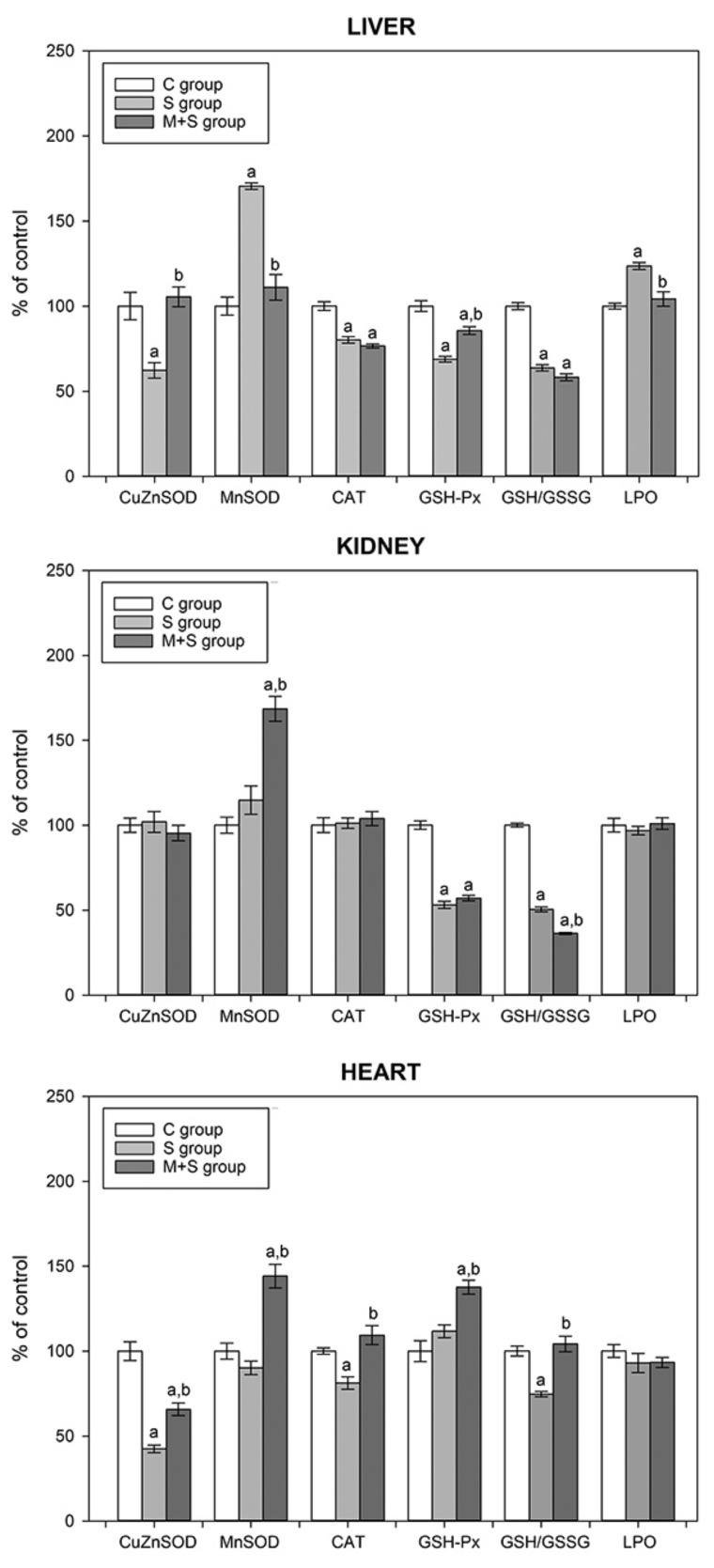
The tissue activities of liver copper-zinc superoxide dismutase (CuZnSOD), manganese superoxide dismutase (MnSOD), catalase (CAT) and glutathione peroxidase (GSH-Px), and tissue glutathione redox ratio (GSH/GSSG) and lipid peroxidation level (LPO) in liver, kidney and heart of rats of control (C), sepsis (S) and meldonium + sepsis (M + S) groups. The data are calculated and graphically presented as the percent of control (mean ± standard error). The number of animals per experimental group: *n* = 8. Minimal significant level: *p* < 0.05. Significantly different: ^a^ with respect to the C group; ^b^ with respect to the S group.

As seen in Figure 2A and Figure 3, the S group exhibited a hepatic increase in p-NF-κB and Nrf2, followed by an MnSOD activity increase, and a decrease in the activity of CuZnSOD, CAT and GSH-Px. This reciprocal pattern of enzyme activation could be explained by the reduced need for CuZnSOD activity since the increased MnSOD activity neutralizes the respiratory-chain-derived superoxide anion radicals and efficiently prevents oxidative stress leakage from mitochondria to the cytoplasm [68]. Additionally, this promotes mitochondria as the primary source of hepatic ROS in LPS sepsis. A reduction in CuZnSOD activity lowers the H_2_O_2_ production and consequently decreases the activity of both CAT and GSH-Px. This decrease in the GSH-Px activity may explain the rise in lipid peroxidation observed in the S group, since GSH-Px, besides its role in H_2_O_2_ conversion, also reduces lipid peroxides [69]. Therefore, the increased level of lipid peroxidation, together with a decrease in GSH/GSSG ratio, strongly suggests increased oxidative stress in the LPS-induced sepsis. In the M + S group, certain changes in the liver antioxidant system were opposite to the S group: a decrease in the MnSOD activity was accompanied by an increase in the CuZnSOD and GSH-Px activities and a decrease in the LPO level (Figure 4). It seems that meldonium reduced overall hepatic oxidative stress, which corresponds well with the hepatic decrease in NF-κB activation. However, this is not in line with the observation of no changes in the hepatic Nrf2 level (Figure 3A). It should be noted that increased oxidative stress in sepsis, and its amelioration by meldonium, are fully in line with the liver histology findings presented in Table 2.

Kidneys exhibited no changes in most of the investigated parameters, except for GSH-Px and GSH/GSSG, whose activity decreased in the S group of animals (Figure 4). GSH-Px is an enzyme that is highly expressed in kidneys, with a protective role against different renal diseases [70]. Its renal overexpression has been shown to reduce oxidative stress in aged mice [71], with the plasma and urine levels being substantially lower in diabetic glomerulosclerosis [72]. Since the GSH-Px action depends on the GSH availability, a decrease in the GSH-Px activity may be explained by shifting the GSH/GSSG ratio in GSSG’s favour. Additionally, a decreased GSH/ GSSG ratio also favours the increased oxidative stress in the S group. As both a nucleophile and a reductant, GSH can directly react with various electrophilic or oxidizing species, preventing them from reacting with critical cellular constituents [73]. Therefore, a decrease in the GSH/GSSG ratio represents a weakening of the antioxidative potential. In the kidneys of the M + S group, meldonium induced a strong increase in the MnSOD activity, followed by an additional decrease in GSH/GSSG renal ratio (Figure 4). It is contradictory that an increase in the MnSOD, which supports the antioxidant defence, was accompanied by a decrease in the GSH/GSSG ratio, which is interpreted as a worsening of oxidative stress.

Interestingly, as opposed to the liver, a rise in the renal p-NF-κB 65 level (Figure 3B) was not accompanied by the MnSOD activity increase. This further underlines the importance of interplay between Nrf2 and NF-kB in the regulation of cellular redox pathways [74]. In summary, LPS sepsis worsened the renal oxidative stress, without clear evidence of meldonium’s protective action, which is in line with the histological findings (Table 2).

Sepsis decreased the cardiac GSH/GSSG ratio, together with the CuZnSOD and CAT activity level, evidencing the oxidative stress increase (Figure 4). Pre-treatment with meldonium restored heart antioxidant defence, judged by the GSH/GSSG ratio and increase in CuZnSOD, MnSOD, CAT and GSH-Px activities. As opposed to liver and kidney, in heart of the M + S group, a strong increase in p-NF-κB 65 level was detected (Figure 3C), implying its importance in the regulation of heart antioxidative defence. It should be emphasized that the meldonium antioxidative effect in the heart corresponds well with the serum Troponin T decrease seen in the M + S group (Table 1), but is not in line with the histology data showing a worsening of heart histology (Table 2).

When the tissue antioxidative potential in the two models of sepsis is compared, both similarities and differences may be observed. Regardless of the model, oxidative stress increased in the liver. However, in the case of FIP, this was only observed at the LPO level [5]. On the contrary, only in the LPS model did meldonium induce a decrease in the LPO level, an effect that was absent in the FIP model. Two models also shared the same pattern of changes in the renal antioxidative status: sepsis worsened the renal oxidative stress, without clear evidence of meldonium exerting its protective potential. Nevertheless, the major difference was observed in the heart. In the FIP model, upon examining the antioxidative status of the heart, we observed no differences between sepsis alone and sepsis in rats pre-treated with meldonium. This was not the case with the LPS-induced sepsis, in which the two septic groups showed a different response regarding the antioxidative potential. In addition, a range of changes in the measured parameters was much broader in the LPS model. In summary, both models of sepsis induction show that the liver endured the largest negative impact, whereas meldonium exerted protective effects in the heart.

Given that sepsis is characterized by both systemic and organ-specific metabolic disturbances [75], we performed an extensive lipidomic analysis (Table 3). As discussed below, the presented lipidomic results are different in comparison with those observed in the FIP model [5], suggesting different patterns of energy production in the two models of sepsis induction.

In the FIP model, a similar pattern of changes was present in all three tissues: an increase in carnitine, glycerol, lactate and total FFAs concentration was observed in the S group, whereas the M + S group exhibited a decrease in these parameters [5]. In addition, glucose concentration was increased in the liver and kidneys of the S group and decreased in the M + S group. We then proposed that, in sepsis, all available energy resources are mobilised, which strengthens the hypothesis of the “septic auto-cannibalism” [76,77]. The decrease in tissue carnitine, glycerol and total FFAs in the M + S group was explained through well-described meldonium effects. Namely, meldonium lowers the L-carnitine concentration by preventing its synthesis through the inhibition of γ-butyrobetaine hydroxylase activity and by preventing its renal reabsorption [78,79]. By disturbing the L-carnitine synthesis, meldonium compromises FFAs β-oxidation and shifts energy production to glycolysis. This explains the observed decrease in the liver and kidney glucose concentration. In addition, this meldonium-induced metabolic shift was followed by a lactate decrease, implicating ATP production through the substrate-level phosphorylation, as previously reported in both liver [6] and kidney [7] models of ischemia/reperfusion injury.

In the LPS-induced sepsis, liver and kidney exhibited similar changes to those observed in the FIP model. Namely, in these tissues, carnitine, TGAs, glycerol, lactate and total FFAs increased and glucose decreased, suggesting an overall increased lipolysis [80] (Table 3). In the heart, the effects of sepsis were somewhat different: carnitine, TGAs and lactate increased, whereas glycerol and total FFAs decreased. As opposed to liver and kidney, it seems that, in the heart, LPS sepsis shifts energy production to glycolysis, as judged by a 2-fold increase in the heart glucose concentration.

However, the main differences in comparison to the FIP model were detected in the M + S group (Table 3). In both liver and kidney, meldonium caused a decrease in TGAs, but an increase in carnitine, glycerol and total FFAs. It seems that, in these two tissues, the expected effects of meldonium were absent, leading to an increase in both TGAs lipolysis and FFAs β-oxidation. Accordingly, a decrease in glucose tissue concentration was not observed, suggesting the absence of a meldonium-induced shift to glycolysis. In the heart, however, the results of the lipidomics analysis were different in comparison to liver and kidney, since meldonium increased carnitine and glucose, and decreased TGAs, glycerol and total FFAs. It seems that, in the heart, meldonium reduces the lipide catabolism, promoting glycolysis as the salvatory energy production pathway, similar to the effects of the LPS-induced sepsis alone.

Sympathoadrenal overstimulation is one of the key players involved in sepsis-induced organ injury [81]. Our results showed that sepsis induced a significant decrease in both noradrenaline and adrenaline adrenal content (by 15% and 30%, respectively; Table 4). These changes are result of increased catecholamine discharge from the adrenal gland, as proved by their 2-fold serum increase. Meldonium pre-treatment had no effect on noradrenaline and adrenaline adrenal content or adrenaline serum concentration. However, meldonium decreased the serum noradrenaline level, returning it to the control level. Therefore, it can be concluded that, in LPS sepsis, both catecholamines are equally important for energy production, while in the meldonium-supplemented group, this role primarily belongs to adrenaline. A decrease in noradrenaline serum concentration in the M + S group may explain a decrease in serum TGAs, glycerol and glucose with respect to the S group.

When the LPS and FIP models are compared, two major differences are evident. In the FIP, sepsis alone slightly decreased adrenaline and slightly increased noradrenaline serum concentration [5], suggesting much weaker sympathoadrenal overstimulation. In contrast, LPS caused a 2-fold increase in both catecholamines. Secondly, in the FIP model, meldonium did not change the serum level of adrenaline, whereas it further reduced the serum level of noradrenaline. Hence, the increased lipid catabolism in the LPS sepsis, and its decrease in the FIP-induced sepsis, may be due to the opposite pattern of sympathoadrenal overstimulation.

Table 4 shows that a more than 3-fold increase in serum glycerol was detected in the S group, followed by a 50% increase in glucose and a 20% decrease in FFAs. Since this massive efflux of glycerol was not accompanied by a parallel secretion of FFAs, it can be attributed to the non-lipolytic origin. Recent studies suggest that white adipocytes synthesize and secrete glycerol to dispose of the excess glucose [82]. In this way, white adipose tissue helps prevent the overall negative effects of hyperglycaemia [82], which could explain the changes observed in the S group. In addition, 3C fragments such as glycerol present ready-to-use energy substrates owing to the easy uptake by tissues in an insulin-independent manner [83,84]. In a healthy heart, the FFAs β-oxidation represents the main substrate for ATP production, even though virtually all substrates partake in the process [85]. If the oxygen availability is compromised, e.g., in ischemia [86], myocardial FFAs oxidation decreases, switching energy production to other substrates, such as glucose and lactate [87], resulting in decreased O_2_ consumption. This control of the glucose/FFAs balance seems to be a key regulatory factor in the heart energy balance under ischemic conditions [88]. As previously hypothesized, the decreased ability of tissues to use oxygen despite its availability suggests the presence of a sort of metabolic ischemia in sepsis due to the impaired energy homeostasis [14]. Hence, the present changes in serum glycerol, glucose and lactate concentrations (Table 4) support our previous conclusion regarding the LPS-induced shift to glycolysis as the salvatory energy production pathway in the heart. The reciprocal pattern of changes in serum glycerol and glucose concentration between the M + S group and S group (Table 4) may indicate the cumulative effect of sepsis and meldonium regarding the myocardial energy production. Namely, the FFAs consumption, already corrupted by the sepsis alone, is further compromised by meldonium, which leads to a more intense use of glucose and glycerol as alternative sources of energy. This results in a decrease in glucose and glycerol in both the serum and heart of the M + S group (Table 3 and Table 4).

A similar pattern of changes of inositol concentration in both serum and tissues, e.g., an increase in the S group and a decrease in the M + S group, present an important finding. Inositol-requiring enzyme 1 (IRE1), which is an endoplasmic reticulum (ER) resident transmembrane protein, mediates the ER stress-signalling pathway in various diseases, including LPS-induced sepsis [89,90,91]. Therefore, an increase in inositol in tissue and serum of the S group indicates increased ER stress, whereas the observed decrease in the M + S group indicates its meldonium-induced reduction. In addition, IRE1-mediated modulation of the NF-κB activation [92] may explain the presented findings in the liver and kidney: an increase in NF-κB p65 in the S and a decrease in the M + S group (Figure 3A,B).

**Figure 5:**
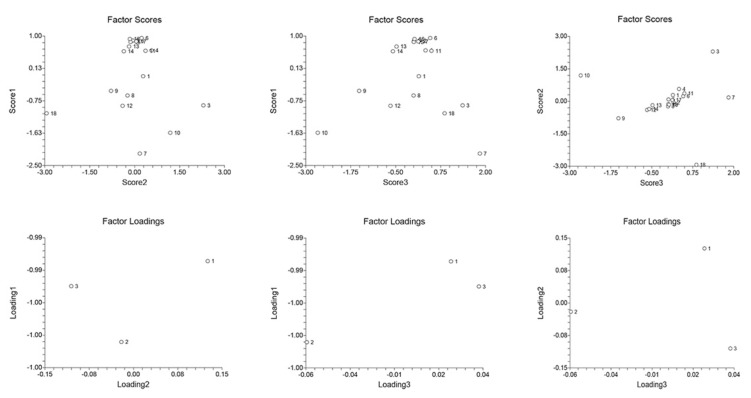
Liver PCA analysis. FFAs abbreviations: 1—C14:0 (myristic); 2—C15:0 (pentadecylic); 3—C16:0 (palmitic); 4—C16:1 (palmitoleic); 5—C17:0 (margaric); 6—C17:1 (heptadecenoic); 7—C18:0 (stearic); 8—C18:1c (oleic); 9—C18:1t (elidic); 10—C18:2c+t (linoleic/linolelaidic); 11—C18:3n3c+t (linolenic); 12—C20 (arachidic); 13—C20:1 (gondoic); 14—C20:2 (eicosadienoic); 15—C20:3n3 (dihomo-α-linolenic, ALA); 16—C20:3n4 (dihomo-γ-linolenic); 17—C20:3n6 (eicosahexaenoic); 18—C22:6 (docosahexaenoic, DHA). See Supplemental Material for more details.

**Figure 6:**
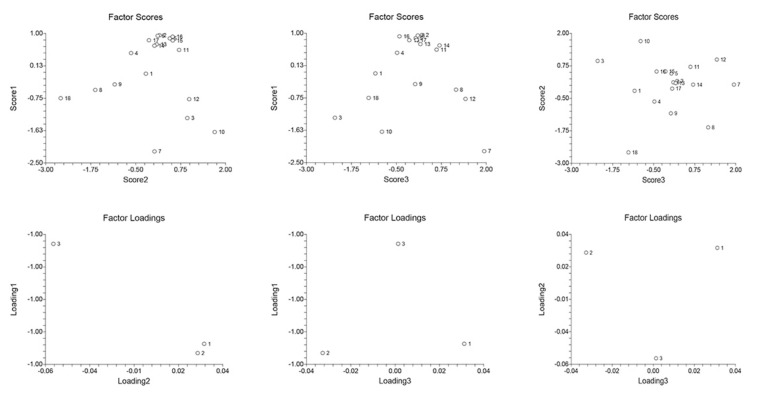
Kidney PCA analysis. FFAs abbreviations: 1—C14:0 (myristic); 2—C15:0 (pentadecylic); 3—C16:0 (palmitic); 4—C16:1 (palmitoleic); 5—C17:0 (margaric); 6—C17:1 (heptadecenoic); 7—C18:0 (stearic); 8—C18:1c (oleic); 9—C18:1t (elidic); 10—C18:2c+t (linoleic/linolelaidic); 11—C18:3n3c+t (linolenic); 12—C20 (arachidic); 13—C20:1 (gondoic); 14—C20:2 (eicosadienoic); 15—C20:3n3 (dihomo-α-linolenic, ALA); 16—C20:3n4 (dihomo-γ-linolenic); 17—C20:3n6 (eicosahexaenoic); 18—C22:6 (docosahexaenoic, DHA). See Supplemental Material for more details.

**Figure 7:**
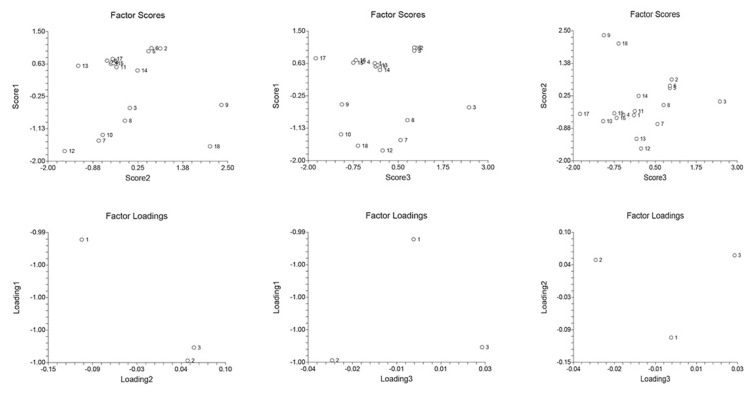
Heart PCA analysis. FFAs abbreviations: 1—C14:0 (myristic); 2—C15:0 (pentadecylic); 3—C16:0 (palmitic); 4—C16:1 (palmitoleic); 5—C17:0 (margaric); 6—C17:1 (heptadecenoic); 7—C18:0 (stearic); 8—C18:1c (oleic); 9—C18:1t (elidic); 10—C18:2c+t (linoleic/linolelaidic); 11—C18:3n3c+t (linolenic); 12—C20 (arachidic); 13—C20:1 (gondoic); 14—C20:2 (eicosadienoic); 15—C20:3n3 (dihomo-α-linolenic, ALA); 16—C20:3n4 (dihomo-γ-linolenic); 17—C20:3n6 (eicosahexaenoic); 18—C22:6 (docosahexaenoic, DHA). See Supplemental Material for more details.

The principal component analysis (PCA) was applied to explore the FFAs’ profile across tissues and experimental groups, (Figure 5, Figure 6 and Figure 7). As seen in Figure 5, C16:0 and C22:6 were predominant FFAs in the liver of the S group (factor scores 2.297 and 2.938, respectively). It is known that palmitic acid negatively correlates with the extent of inflammation [93]. This correlation is probably due to the induction of trained immunity, which is in charge of inflammation and infection [94]. Interestingly, DHA concentration not only increased in the liver of the S group, but further increased in the M + S group. Docosahexaenoic acid is of great importance when fighting inflammation, especially in the heart [95] and brain [96]. It has been suggested that, in mammals, rates of DHA synthesis from α-linolenic acid (18:3n-3, ALA) are low relative to dietary intake, since increased dietary ALA intake is not followed by an increase in DHA plasma concentration [97]. However, an alternative mechanism of DHA synthesis was also proposed [98,99]. Increased DHA in the liver may be a part of the cell survival strategy under septic conditions. This further increase in the liver of the M + S group may be explained by a link to the carnitine availability. It has been proposed that carnitine deficiency might cause DHA deficiency [100], thus representing a common denominator in various oxidative phosphorylation and FFAs β-oxidation disorders [101]. Therefore, the increased DHA concentration in the M + S group could be explained by the simultaneous increase in liver carnitine (Table 3). In the liver FFAs profile of the M + S group, the most influential FFA was C18:2c+t, with a factor score of 2.6637. Linoleic acid is already recognized for its detrimental effects in sepsis, with the concentration rise associated with the fatal outcome of sepsis [102]. Hence, the fact that its concentration was increased in the liver of the S group, but remained unchanged in the M + S group, indicates the protective effect of meldonium.

In kidneys of the S group, similar to that of the liver, C22:6 was the FFA with the highest factor score (Figure 6). In the M + S group, C16:0 and C18:0 contributed the most to the kidneys’ FFAs profile (factor scores of 2.206 and 1.945, respectively). It can be assumed that in both the liver and kidney, palmitic and docosahexaenoic acid are of great importance in coping with sepsis, since their concentrations increased in the S group and further increased in the M + S group. As for stearic acid, it is known that its concentration is decreased in the blood of patients suffering from chronic renal disease, with its reduction associated with a declining renal function [103]. Hence, the 50% increase in C18:0 in the M + S group compared to the S group suggests meldonium-induced renal protection.

In the S group, C22:6 and C18:1c were contributed the most to the FFA profile of the heart (factor score 2.058 and 2.344, respectively, Figure 7). This is in line with a finding that mice fed with oleic acid had a higher survival rate following a caecal ligation and puncture-induced sepsis [104]. These protective properties of oleic acid probably include upregulation of the carnitine palmitoyltransferase 1A [105]. Therefore, an increase in both L-carnitine and C18:1c in all three tissues (Table 3) may represent a cell-survival strategy under the septic conditions. In the M + S group, the FFA with the highest factor score (2.2475) in the heart was C16:0, as was the case in the kidney. Summarising the results of the PCA analysis, we conclude that palmitic and docosahexaenoic acid were predominant among both tissues and experimental groups.

Finally, a question arises whether the present effects of meldonium are sepsis-independent, i.e., whether meldonium causes the same effects in sham animals. We did not include a sham+meldonium experimental group in either the FIP or LPS model, since this was included in our previous experiments. Namely, in both liver [6] and renal [7] models of ischemia/reperfusion, we demonstrated that meldonium exerted no harmful effects on sham animals. Therefore, we conclude that the present results are the outcome of meldonium’s and sepsis joint effects.

## 3. Materials and Methods

### 3.1. Animals and Treatments

All animal procedures were performed in compliance with the ARRIVE guidelines and Directive 2010/63/EU. Following the national legislation, all animal procedures were approved by the Veterinary Directorate of the Ministry of Agriculture, Forestry and Water Management, license number 323-07-11574/2021-05.

Sprague-Dawley strain (Rattus norvegicus) male rats weighing 240.17 ± 3.81 g were used for the experiment. The animals were acclimated to 22 ± 1 °C and maintained under a 12 h light/dark regime. The rats were randomly divided into three groups and housed two per cage for four weeks with ad libitum access to a standard diet (Veterinary Institute, Subotica, Serbia) and tap water (with or without meldonium).

Rat groups were as follows (Table 5): control sham group of 8 animals that drank tap water for four weeks and then received a saline injection (C group); a septic group of 8 animals that drank tap water without meldonium for four weeks and then received an LPS intraperitoneal injection (S group); and the meldonium-septic group of 8 animals that drank tap water with meldonium for four weeks and then received an LPS intraperitoneal injection (M + S group). The animal weights per group was 236.38 ± 8.12 g, 241.13 ± 6.48 g, and 243.22 ± 5.62 g for the C, S and M + S group, respectively. At the end of the experiment, animals were decapitated for serum and tissue collection.

Meldonium (3-(2,2,2- trimetilhidrazinijum) propionate; THP; MET-88, manufacturer Shenzhen Calson Bio-Tech Co., Ltd., Shenzhen, China) was dissolved in tap water in concentrations ranging from 2 to 3 mg/mL. Depending on the water intake, meldonium concentrations in the water were adjusted weekly to achieve a consumption of around 300 mg/kg b.m./day. Based on the four-week measurement, the meldonium consumption in M + S group was 280.48 ± 3.36 mg/kg b.m./day.

On the day of sepsis initiation, LPS was dissolved in 5 mg LPS/1 mL saline concentration and given as 1mL/1 kg of animal body mass. Animals from control groups received a sham injection with saline only (1 mL of saline/1 kg b.m.). After the sepsis initiation, animals’ rectal temperature monitored each hour.

### 3.2. Serum and Tissue Collection

Rats were euthanized by decapitation at any given timepoint between 8 and 9 h after initiation of sepsis [106], and blood and tissue samples were collected immediately after euthanasia.

Blood was collected and incubated at room temperature for 45 min to allow for clot formation. Then, clots were removed by centrifugation at 2000× *g* for 10 min at 4 °C. The resulting supernatant was immediately transferred into a clean polypropylene tube using a Pasteur pipette [107].

The animal’s liver, kidney and heart were isolated and dissected within 3 min, and washed with ice-cold 155 mmol NaCl. One part of each tissue was placed in formaldehyde for further histological analysis. The serum and the rest of the tissue samples were stored at −80 °C for further analysis.

### 3.3. Biochemical Analysis

#### 3.3.1. Serum Analysis

Activities of ALT, AST and AP in serum were measured by Roche Cobas C501 automated analyser (Roche Diagnostics, Mannheim, Germany), using ALTL, ASTL and ALP2L reagent cassette.

Cardiac Troponin T level was measured with a highly sensitive assay based on electrochemiluminescence technology, using the Roche Cobas e601 automated analyser (Roche Diagnostics, Mannheim, Germany).

The concentrations of proinflammatory cytokine TNFα in the serum of study rats were determined by commercial ELISA kits (Elabscience, Wuhan, China), according to the manufacturer’s instructions. The optical densities were measured at 450 nm using a Multiscan FC microplate reader (Thermo Scientific, Waltham, MA, USA), and concentrations were calculated according to the obtained four-parameter logistic standard curve.

#### 3.3.2. Tissue Analysis of L Carnitine, Lactic Acid, Glucose, Glycerol and Inositol

Tissue samples (50 mg) were mixed with 2 mL of ultra-pure water and pulverized with a tissue grinder. After extraction in an ultrasound bath termostated at 0 °C, samples were centrifuged at 12,000 rpm. The supernatant was transferred in a 10 mL normal flask and diluted with ultra-pure water to the mark. The sample solutions were kept at −80 °C till analysis.

*Tissue carnitine determination*: L-Carnitine standard was obtained from Sigma Aldrich. A total of 10 mg of L-carnitine standard was weighted in 10 mL normal flask and diluted with ultra-pure water to mark. Ascending thin-layer chromatography was performed on RP-18 silica (Art. 5559, Merck, Darmstadt, Germany), used as an adsorbent. The chromatograms were developed using an acetonitrile-water binary mixture with a 1:1 volume ratio. The classical chromatographic chamber (Camag, Muttenz, Switzerland) was filled with 3 mL of mobile phase made of components of an analytical grade of purity. The chamber was saturated with solvent for 30 min. All experiments were performed at ambient temperature. CAMAG Linomat 5 was applied to plates, with 2 µL aliquots of previously prepared and defrosted aqueous solutions. The plates were scanned by CAMAG TLC Scanner 3 at 260 nm, and the obtained chromatograms were analyzed by winCATS software version 1.4.2.8121 (Camag, Muttenz, Switzerland).

*Tissue lactic acid determination*: the lactic acid standard was purchased from Sigma Aldrich. Ion chromatography was used to assay the appearance and quantification of lactic acid. For that purpose, a Dionex ICS-3000 chromatographic set-up (Dionex, Sunnyvale, CA, USA) consisted of a single pump, conductivity detector (ASRS ULTRA II (4 mm) (P/N 061561), recycle mode), eluent generator (potassium hydroxide (KOH) (P/N 058900)) with Chromeleon^®^ Chromatography Workstation with Chromeleon 6.7.2 Chromatography Management Software (Thermo Scientific, Waltham, MA, USA) was employed. All the separation was performed on IonPac AS15 Analytical, 4 × 250 mm (P/N 053940) and IonPac AG15 Guard, 4 × 50 mm (P/N 053942) column. Mobile phase flow rate was set to 1000 mL/min, while the concentration of potassium hydroxide was changeable to achieve the following gradients: 0–15 min. 10 mM KOH; 15–25 min. 10–45 mM KOH; 2526 min. 45 mM KOH; 26–31 min. 45–10 mM KOH; and 31–36 min. 10 mM KOH. The column temperature was termostated at 30 °C, conductivity cell temperature was 35 °C, suppressor current was 134mA and the backpressure was ~2200 psi.

*Tissue glucose and glycerol determination*: The glucose standard was purchased from Tokyo Chemical Industry, TCI Europe, (Zwijndrecht, Belgium), while glycerol standard was obtained from Sigma-Aldrich (Steinheim, Germany). Sodium hydroxide and sodium acetate trihydrate were obtained from Merck (Darmstadt, Germany). All aqueous solutions were prepared using ultrapure TKA deionized water. A standard solution of glucose was prepared in ultrapure water at 100 ng/mL concentration. Calibration standards were prepared from stock solution by dilution with ultrapure water. The quality control mixture used to monitor instrument performance was prepared by diluting standard to concentrations of 20 ng/mL. Chromatographic separations were performed using Dionex ICS 3000 DP liquid chromatography system (Dionex, Sunnyvale, CA, USA) equipped with a quaternary gradient pump (Dionex, Sunnyvale, CA, USA), a Carbo Pac^®^PA100 pellicular anion-exchange column (4 × 250 mm) (Dionex, Sunnyvale, CA, USA) at 30 °C. The mobile phase consisted of the following linear gradients (flow rate, 0.7 mL/min): 0–5 min. 15% A, 85% C; 5.0–5.1 min. 15% A, 2% B, 83% C; 5.1–12 min. 15% A 2% B, 83% C; 12–12.1 min. 15% A, 4% B, 81% C; 12.1–20 min. 15% A, 4% B, 81% C; 20–20.1 min. 20% A, 20% B 60% C; 20.1–30 min. 20% A, 20% B, 60% C; where A was 600mM sodium hydroxide, B was 600 mM sodium acetate and C was ultrapure water. Previously, the analysis system was preconditioned at 15% A and 85% C for 15 min. Each sample (25 µL) was injected with an ICS AS-DV 50 autosampler (Dionex, Sunnyvale, CA, USA). The electrochemical detector consisted of gold as a working electrode and Ag/AgCl as a reference electrode.

*Tissue inositol determination:* The standard myo-inositol was purchased from Sigma Aldrich. Ion chromatography was used to assay the myo-inositol. The analysis was performed on Dionex ICS-3000 chromatographic system (Dionex, Sunnyvale, CA, USA) equipped with a quaternary pump and pulse amperometry detector (PAD). The eluent was a binary mixture of ultra-pure water/100 mM sodium hydroxide and Chromeleon 6.7.2 Chromatography Management Software (Thermo Scientific, Waltham, MA, USA). All the separation was performed on CarboPac PA 10 Analytical, 4 × 250 mm (P/N 046110) and on CarboPac PA 10 Guard, 4 × 50 mm (P/N 046115) column. The mobile phase flow was set to 1.000 mL/min, while the concentration of sodium hydroxide was changeable to achieve the following gradients: 0–15min. 10mM NaOH; 15–25 min. 10–45mM NaOH; 25–26 min. 45mM NaOH; 26–31 min. 45–10mM NaOH; and 31–36 min. 10mM NaOH. The column was termostated at 30 °C. The PAD detector was composed of gold as a working electrode and glass as referent electrode.

#### 3.3.3. Lipidomics Tissue Preparation

Tissue samples (150 mg) were mixed with 2 mL of chloroform/methanol (2/1, *v*/*v*) and pulverized with a tissue grinder. After extraction in an ultrasound bath termostated at 0 °C, 1 mL of 0.9% solution of NaCl was added. The samples were centrifuged at 12,000 rpm. The supernatant was transferred to a test tube and evaporated in a stream of nitrogen. The solid residues were dissolved in 3 mL of hexane, and 1 mL was kept for further analysis, whereas 2 mL were hydrolyzed with 1 mL of 2 M KOH solution in methanol. The mixture was put in an ultrasonic bath for 2 min at 70 °C. The excess potassium hydroxide was neutralized with 2 M HCl solution in methanol. To extract fatty acids, 3 mL of hexane was added to the mixture. The organic layer (hexane) was collected and evaporated. The solid residues were resolved with 1 mL of hexane fortified with 50 µL of previously made solution of methyl nonadecanoate (C19:0) (internal standard; ISTD) in hexane and kept at −80 °C until analysis.

#### 3.3.4. Tissue Fatty Acids Determination

The FFAs analysis in the obtained samples was performed at Focus GC coupled with a PolarisQ mass spectrometer (Thermo Fisher, Waltham, MA, USA). The carrier gas was helium (1 mL/min) and the injected volume of the sample was 1 µL. The temperature program of the oven was as follows: the initial temperature 50 °C (1 min), then 25 °C/min to 200 °C and, immediately afterwards, 3 °C/min to 230 °C (held for 18 min). The injector was in split mode (50:1), while the temperatures of the injector, transfer line and ion source were 250 °C, 260 °C and 260 °C, respectively. The concentration of 18 fatty acids was investigate: FFAs abbreviations: C14:0—myristic; C15:0—pentadecylic; C16:0—palmitic; C16:1—palmitoleic; C17:0—margaric; C17:1—heptadecenoic; C18:0—stearic; C18:1c—oleic; C18:1t—elidic; C18:2c+t—linoleic/linolelaidic; C18:3n3c+t—linolenic; C20—arachidic; C20:1—gondoic; C20:2—eicosadienoic; C20:3n3—dihomo-α-linolenic; C20:3n4—dihomo-γ-linolenic; C20:3n6—eicosahexaenoic; C22:6—docosahexaenoic. Under applied chromatographic conditions cis- and trans- forms of C18:2 and C18:3n3 coelute at the same retention time, so their concentrations were calculated as the sum.

#### 3.3.5. Tissue Triglycerides Determination

The TGAs analysis was performed on Camag TLC Scanner 3. A total of 2 µL of lipid extract was applied to 20 × 20 cm HPTLC silica gel plates (Art. 105641, Merck, Darmstadt, Germany) as a 6 mm band using Automatic TLC sampler 4 (ATS4, CAMAG, Muttenz, Switzerland). The ascending chromatography was performed in CAMAG twin-trough chamber using four mobile phases: chloroform:methanol:acetic acid (90:10:1, *v*/*v*/*v*) up to 25 mm, n-hexane:diethyl-ether:acetone (60:40:5, *v*/*v*/*v*) up to 70 mm, n-hexane:diethyl-ether up to 85 mm and 100% n-hexane up to 90mm. Before analysis, the chambers were saturated for 30 min. After the last mobile phase development, the plates were dried in the dark. Derivatisation was performed by spraying with a mixture of methanol and concentrated sulphuric acid (9/1, *v*/*v*). Derivatized plates were heated at 80 °C on TLC Plate Heater III (CAMAG) until chromatographic zones became visible, followed by scanning in CAMAG TLC scanner 3. TGAs concentration was determined based on the intensity of the standards and samples.

### 3.4. Determination of Oxidative Stress Biomarkers

For oxidative status analysis all samples were homogenized in cold phosphate-buffered saline (1:4 mass/volume) using IKA T 10 Basic Ultra Turrax Homogenizer (IKA^®^-Werke GmbH & Co. KG, Staufen, Germany) and centrifuged for 30 min at 4 °C and 13,000× *g* (Eppendorf microcentrifuge 5417R, Hamburg, Germany). The obtained supernatants were stored at −80 °C until oxidative status analysis. All assays were performed in triplicate.

The level of MDA was assessed according to the method of Gérard–Monnier and co-workers [108]. Solution of 1-methyl-2-phenylindole in a mixture of acetonitrile/methanol was added to the sample. The reaction was started by adding HCl and, upon incubation of the reaction mixture at 45 °C for 40 min, the absorbance was measured at 586 nm on microplate reader (WALLAC 1420-Victor2 Multilabel Counter, PerkinElmer, Waltham, MA, USA). MDA concentration was determined using the corresponding standard curve and expressed in µM.

The Misra and Fridovich method relied on the SOD’s ability to inhibit the conversion of adrenaline to adrenochrome. This was used for total SOD activity assessment [109]. Reactions were monitored at 480 nm, on S-40 Boeco spectrophotometer (Germany). Following the inhibition of CuZnSOD with KCN, MnSOD activity was estimated by the same procedure. CuZnSOD activity corresponds to the difference between total SOD and MnSOD activities. One unit of SOD activity is equivalent to the amount of the enzyme that inhibits 50% of the adrenaline autoxidation. The results are presented as a specific activity of the enzyme in 1 mg of tissue (U/mg).

CAT activity was determined by measuring absorbance decrease at 240 nm [110] on S-40 Boeco spectrophotometer (Germany). The enzymes’ activity corresponds to the decline in absorbance due to H2O2 degradation. One unit of CAT activity is equivalent to the amount of enzyme that leads to 90% decomposition of the substrate in 1ml of reaction mixture for 1 min. CAT activity was displayed as enzyme activity in 1 mg of tissue (U/mg).

Concentrations of GSH and GSSG were assayed by adding samples to Na-phosphate buffer containing ethylenediaminetetraacetic acid (EDTA) and 5,50-dithio-bis-2-nitrobenzoic acid (DTNB). After 5 min, the absorbance was recorded at 412 nm on S-40 Boeco spectrophotometer (Germany) and level of GSH was calculated according to the standard curve. The amount of total glutathione (GSH plus GSSG) was estimated 30 min after adding NADPH and glutathione reductase (GR) to the reaction mixture and measuring the absorbance at the same wavelength. Levels of GSH and GSSG were expressed in µM [111].

GPx activity was estimated as the change in absorbance at 340 nm, measured on WALLAC 1420-Victor2 Multilabel Counter, PerkinElmer, Waltham, MA, USA. This method is based on the NADPH-mediated reduction in GSSG, using GR [112]. GPx activity was proportional to the decline in absorbance and expressed as U/g of tissue.

### 3.5. Determination of Adrenaline and Noradrenaline Content

#### 3.5.1. Determination of Adrenaline and Noradrenaline Content in Serum

Determination of serum catecholamines was commercially performed in BelMedic laboratory, Belgrade, Serbia.

#### 3.5.2. Determination of Adrenaline and Noradrenaline Content in Adrenal Glands

For high-performance liquid chromatography (HPLC) analysis, adrenal glands were dissected and immediately stored at −70 °C. Tissue samples were homogenized in DEPROT solution (1 mg:30 μL) containing 2% ethylene glycol tetra-acetic acid, 0.1 N HClO4 and 0.2% MgCl2, sonicated and centrifuged (30 min, 18,000 rpm, 4 °C). A total of 50 µL of collected supernatants were injected with the autosampler of a Dionex UltiMate 3000 HPLC system (Thermo Scientific, Sunnyvale, CA, USA) equipped with a Acclaim Polar Advantage II (C18, 5 µm, 4.6 mm, 150 mm) HPLC column (Thermo Scientific, Waltham, MA, USA). Chromeleon7 Chromatography Data System (Thermo Scientific, Sunnyvale, CA, USA) was used for instrument control and data acquisition. The mobile phase consisted of 98% ammonium formate buffer (Fisher Scientific, Cambridge, UK, pH 3.6) and 2% methanol (J.T. Baker, Griesheim, Germany), with the flow rate set at 500 μL/min. The electrochemical measurement was set at +850 mV potential and the separation temperature at 25 °C. Noradrenaline (DL-noradrenaline hydrochloride, Sigma-Aldrich) and adrenaline (±)-adrenaline hydrochloride, Sigma-Aldrich) standard solutions were made from the stock standard solution (1 mg/mL of noradrenaline in methanol) in DEPROT, with concentration range 0.5–50 μg/mL.

### 3.6. Western Immunoblot Analysis

Liver, kidney and heart tissue samples were homogenized using IKA T 10 Basic Ultra Turrax Homogenizer (IKA^®^-Werke GmbH & Co. KG, Staufen, Germany) in ice-cold RIPA buffer (50 mM Tris-HCl pH = 7.44; 0.1% SDS; 150 mM NaCl; 10 mM EDTA; 10 mM EGTA; 1% NP-40; 0.5% Triton-X) containing protease and phosphatase inhibitors (SigmaFAST protease inhibitor cocktail and Na-orthovanadate, respectively). Homogenates were centrifuged at 10,000× *g* (Sorvall SL-50T, Super T21, Thermo Fisher Scientific) for 20 min at 4 °C. Resulting supernatants were aliquoted and stored at −80 °C.

Protein samples of whole liver, kidney and heart homogenates (20 µg) were separated by 12% SDS-PAGE and transferred to a PVDF membranes (BioRad, USA), blocked in 5% non-fat condensed milk—TBST solution (0.2% Tween 20, 50 mM Tris-HCl pH 7.6, 150 mM NaCl) and incubated overnight, at 4 °C, with different primary antibodies: rabbit polyclonal anti-HMGB1 (1:1000, ab18256, Abcam, UK), rabbit monoclonal anti-phospho-NF-κB p65 (1:1000, Cell Signaling, CS-8242), rabbit polyclonal anti-PGC1α-antibody (1:1000, ab191838; Abcam), mouse monoclonal anti-Bax (1:5000, Santa Cruz Biotechnology, Inc USA, sc-7480), rabbit polyclonal anti-Bcl2 (1:5000, Santa Cruz Biotechnology, Inc USA, sc-492), goat polyclonal anti-Nrf2 (1:500, Santa Cruz Biotechnology, Inc USA, sc-30915) and goat polyclonal β -actin (1:5000, Santa Cruz Biotechnology, Inc USA, sc-1615). Monoclonal mouse anti-GAPDH (1:10,000, Millipore, MAB374) and HRP anti-β actin (1:1000, Abcam, ab49900) were used to confirm equal sample loading.

After rinsed in TBST solution, the blots were incubated with horseradish peroxidase-conjugated secondary antibodies resuspended in TBST: goat anti-mouse IgG HRP (1:10,000, Abcam, ab97240) and goat anti-rabbit IgG HRP (1:30,000, Abcam, ab6721).

The detection of chemiluminescent signals was developed by Clarity Western ECL Substrate (Bio-Rad, Hercules, CA, USA). ChemiDoc-It Imager (Ultra-Violet Products Ltd., Cambridge, UK) was used to capture images. Quantification was performed in the Image J program (version 1.80, National Institutes of Health, USA).

### 3.7. Histology Analysis

Tissue samples from the heart, liver and kidney were fixed in a 10% neutral buffered formalin for ~48, washed in a series of increasing ethanol solutions (70%, 96% and 100%), and immersed in xylene. Afterwards, samples were embedded into paraffin wax, cut into 5-µm-thick sections and stained with Haematoxylin and Eosin (H&E) stain. A minimum of 10 fields of each organ section were analyzed by an Olympus BX43 microscope connected to a Leica ICC50W camera.

Morphological changes in liver tissue were scored according to the Suzuki histological score system, based on the intensity of sinusoidal congestion, vacuolization of hepatocyte cytoplasm and parenchymal necrosis (0—no changes; 1—minimal changes, 2—mild changes, 3—moderate changes, 4—severe changes) [113].

Renal damage was evaluated by renal damage score system, based on the intensity of tubular epithelial cell flattening (TF), brush border loss (BBL), tubular necrosis (TN) and tubular lumen obstruction (TO) (0—no changes; 1—minimal changes, 2—mild changes, 3—moderate changes, 4 and over—severe changes) [114].

Heart tissue was evaluated using the Dallas criteria for the diagnosis of myocarditis, based on the extent of inflammatory infiltration (II) (0—no changes; 1—mild changes, 2—moderate changes, 3—severe changes), and their distribution (1—focal; 2—confluent, 2—mild changes, 3—diffuse) [115].

### 3.8. Statistical Analysis

Differences in investigated parameters between the groups were calculated using one-way ANOVA. When significant differences were found, pairwise comparisons were performed using Holm–Sidak post hoc tests. Survival Analysis was carried out using Kaplan–Meier survival analysis, using the LogRank test to compare multiple curves. A statistical package SIGMAPLOT was used for all the analyses and graphical presentations, except the PCA analysis, which was performed using the NCSS 2004 software package. The level of statistical significance was defined as *p* < 0.05. Where appropriate, analysis results were graphically presented as the percentage of the control (C) group.

## 4. Conclusions

Despite some shared similarities, when it comes to energy production, it is evident that LPS and FIP models of sepsis induction differ greatly. A different profile of sympathoadrenal activation may account for this observation, as this was lacking in the FIP model, whereas in the LPS model it was strong enough to overcome the effects of meldonium. Therefore, choosing an appropriate model of sepsis induction is of great importance, especially if energy homeostasis is the main focus of the study. Even when differences in the experimental design of the two models are acknowledged, the role of different patterns of energy production cannot be ignored. On that account, our results no only underline not only the importance of uninterrupted energy production in sepsis, they also call for much-needed revisions of the current recommendations for its treatment.

## Figures and Tables

**Table 1:** The serum aspartate aminotransferase (AST), alanine aminotransferase (ALT) and alkaline phosphatase (ALP) activities (IU/L), and Troponin T (ng/L), urea (mmol/L), creatinine (mmol/L) and TNFα (pg/mL) concentrations in rats from control (C), sepsis (S) and meldonium + sepsis (M + S) groups. The number of animals per experimental group: *n* = 8. The data are given as mean ± standard error of the mean. Minimal significant level: *p* < 0.05. Significantly different: ^a^ with respect to the C group; ^b^ with respect to the S group.

	C Group	S Group	M + S Group
AST	271.15 ± 13.93	288.10 ± 18.12 ^a^	212.83 ± 4.11 ^a,b^
ALT	65.18 ± 3.88	91.71 ± 2.32 ^a^	62.45 ± 1.62 ^b^
ALP	122.25 ± 4.49	164.50 ± 5.49 ^a^	166.67 ± 3.86 ^a^
Troponin T	31.25 ± 1.93	124.28 ± 2.99 ^a^	64.66 ± 1.62 ^a,b^
Urea	5.19 ± 0.13	11.76 ± 0.42 ^a^	7.83 ± 0.35 ^a,b^
Creatinine	45.03 ± 0.67	50.69 ± 1.62	47.88 ± 0.73
TNFα	11.32 ± 1.73	34.13 ± 2.57 ^a^	17.60 ± 2.11 ^a,b^

**Table 2:** Tissue histological score in liver, kidney and heart of rats of control (C), sepsis (S), and meldonium + sepsis (M + S) groups: Liver Suzuki histological score (0–4) based on the sinusoidal congestion, vacuolization of hepatocyte cytoplasm and parenchymal necrosis (0—no changes; 1—minimal changes, 2—mild changes, 3—moderate changes, 4—severe changes); Renal histological score (0–4) based on the tubular epithelial cell flattening (TF), brush border loss (BBL), tubular necrosis (TN) and tubular lumen obstruction (TO) (0—no changes; 1—minimal changes, 2—mild changes, 3—moderate changes, 4 or above—severe changes); Heart histological score (Dallas criteria) based on the extent of inflammatory infiltration (II) (0—no changes; 1—mild changes, 2—moderate changes, 3—severe changes) and their distribution (1—focal; 2—confluent, 2—mild changes, 3—diffuse).

Liver	C Group	S Group	M + S Group
Congestion	1	2.71	1.25
Vacuolization	0.50	2.29	1.13
Necrosis	0.50	2.14	1.13
**Kidney**	**C group**	**S group**	**M+S group**
TF	4.25	7.57	6.38
BBL	4.13	12.29	11.50
TN	1.25	8.57	7.88
TO	0.00	9.86	6.38
**Heart**	**C group**	**S group**	**M+S group**
II	0.00	0.25	0.88
Distribution	0.00	0.25	0.63

**Table 3:** Liver, kidney and heart lipidomics in rats of control (C), sepsis (S) and meldonium + sepsis (M + S) groups. L carnitine, triglycerides (TGAs), glycerol, total free fatty acid content (FFAs), glucose, lactate and inositol are expressed in mg/g, whereas concentrations of individual FFAs are expressed in μg/g. FFAs abbreviations: C14:0—myristic; C15:0—pentadecylic; C16:0—palmitic; C16:1—palmitoleic; C17:0—margaric; C17:1—heptadecenoic; C18:0—stearic; C18:1c—oleic; C18:1t—elidic; C18:2c+t—linoleic/linolelaidic; C18:3n3c+t—linolenic; C20—arachidic; C20:1—gondoic; C20:2—eicosadienoic; C20:3n3—dihomo-α-linolenic (ALA); C20:3n4—dihomo-γ-linolenic; C20:3n6—eicosahexaenoic; C22:6—docosahexaenoic (DHA). The number of animals per experimental group: *n* = 8. The data are given as mean ± standard error of the mean. Minimal significant level: *p* < 0.05. Significantly different: ^a^ with respect to the C group; ^b^ with respect to the S group. The number of animals per experimental group: *n* = 8. The data are given as mean ± standard error of the mean. Minimal significant level: *p* < 0.05. Significantly different: ^a^ with respect to the C group; ^b^ with respect to the S group.

Liver	C Group	S Group	M + S Group
L carnitine	0.575 ± 0.006	0.625 ± 0.005 ^a^	0.682 ± 0.004 ^a,b^
TGAs	1.054 ± 0.015	1.224 ± 0.005 ^a^	0.644 ± 0.010 ^a,b^
Glycerol	2.269 ± 0.014	2.665 ± 0.015 ^a^	2.908 ± 0.008 ^a,b^
Total FFAs	2.216 ± 0.137	2.487 ± 0.090 ^a^	2.880 ± 0.90 ^a,b^
Glucose	1.217 ± 0.002	1.091 ± 0.003 ^a^	1.078 ± 0.028 ^a^
Lactate	2.626 ± 0.021	2.777 ± 0.021^a^	2.419 ± 0.009 ^a,b^
Inositol	0.610 ± 0.002	1.476 ± 0.013 ^a^	1.169 ± 0.029 ^a,b^
C14:0	138.091 ± 0.849	147.201 ± 0.530 ^a^	169.343 ± 0.528 ^a,b^
C15:0	18.815 ± 0.116	27.656 ± 0.099 ^a^	39.286 ± 0.091 ^a,b^
C16:0	264.837 ± 1.628	231.321 ± 0.833 ^a^	263.097 ± 0.820 ^b^
C16:1	60.166 ± 0.370	59.043 ± 0.210	60.523 ± 0.189
C17:0	21.291 ± 0.130	29.235 ± 0.105 ^a^	28.149 ± 0.085 ^a^
C17:1	15.813 ± 0.097	16.153 ± 0.059	15.991 ± 0.049
C18:0	388.473 ± 2.389	425.616 ± 1.424 ^a^	496.444 ± 1.547 ^a,b^
C18:1t	191.625 ± 1.179	224.628 ± 0.772 ^a^	255.525 ± 0.797 ^a,b^
C18:1c	164.510 ± 1.013	191.591 ± 0.762 ^a^	236.689 ± 0.738 ^a,b^
C18:2c+t	325.322 ± 2.001	366.350 ± 1.283 ^a^	369.451 ± 1.151 ^a^
C18:3n3c+t	58.821 ± 0.361	58.657 ± 0.211	66.282 ± 0.206 ^a,b^
C20	220.391 ± 1.355	254.474 ± 0.916 ^a^	296.440 ± 0.924 ^a,b^
C20:1	34.304 ± 0.211	52.799 ± 0.189 ^a^	51.979 ± 0.162 ^a^
C20:2	46.596 ± 0.286	70.005 ± 0.253 ^a^	71.599 ± 0.229 ^a^
C20:3n3	21.751 ± 0.133	33.780 ± 0.121 ^a^	35.362 ± 0.110 ^a^
C20:3n4	11.974 ± 0.073	23.725 ± 0.085 ^a^	23.824 ± 0.074 ^a^
C20:3n6	23.334 ± 0.144	30.689 ± 0.110 ^a^	31.024 ± 0.097 ^a^
C22:6	210.238 ± 1.293	275.059 ± 0.990 ^a^	376.264 ± 1.172 ^a,b^
**Kidney**	**C group**	**S group**	**M+S group**
L carnitine	0.623 ± 0.004	0.692 ± 0.001 ^a^	0.730 ± 0.002 ^a,b^
TGAs	0.492 ± 0.006	0.738 ± 0.005 ^a^	0.164 ± 0.002 ^a,b^
Glycerol	0.355 ± 0.002	0.783 ± 0.002 ^a^	0.977 ± 0.009 ^a,b^
FFAs	1152.978 ± 3.261	1330.299 ± 3.053 ^a^	1603.681 ± 7.077 ^a,b^
Glucose	0.732 ± 0.002	0.671 ± 0.003 ^a^	0.687 ± 0.002 ^a^
Lactate	2.462 ± 0.011	3.236 ± 0.009 ^a^	2.932 ± 0.011 ^a,b^
Inositol	0.362 ± 0.002	0.859 ± 0.005 ^a^	0.695 ± 0.002 ^a,b^
C14:0	66.987 ± 0.189	81.855 ± 0.188 ^a^	96.851 ± 0.427 ^a,b^
C15:0	8.126 ± 0.023	10.325 ± 0.023 ^a^	14.555 ± 0.065 ^a,b^
C16:0	138.451 ± 0.391	166.398 ± 0.383 ^a^	185.056 ± 0.817 ^a,b^
C16:1	34.176 ± 0.097	42.641 ± 0.098 ^a^	55.385 ± 0.244 ^a,b^
C17:0	13.323 ± 0.037	16.809 ± 0.039 ^a^	19.824 ± 0.088 ^a,b^
C17:1	8.669 ± 0.024	11.440 ± 0.026 ^a^	15.863 ± 0.070 ^a,b^
C18:0	198.424 ± 0.561	216.048 ± 0.497 ^a^	319.694 ± 1.146 ^a,b^
C18:1t	94.951 ± 0.269	103.939 ± 0.239 ^a^	137.438 ± 0.607 ^a,b^
C18:1c	84.793 ± 0.239	97.285 ± 0.223 ^a^	123.254 ± 0.544 ^a,b^
C18:2c+t	164.795 ± 0.466	190.118 ± 0.438 ^a^	211.336 ± 0.933 ^a,b^
C18:3n3c+t	33.525 ± 0.095	37.895 ± 0.084 ^a^	43.130 ± 0.190 ^a,b^
C20	114.894 ± 0.325	124.500 ± 0.285 ^a^	145.548 ± 0.642 ^a,b^
C20:1	22.631 ± 0.064	28.770 ± 0.061 ^a^	34.097 ± 0.151 ^a,b^
C20:2	25.598 ± 0.073	30.836 ± 0.064 ^a^	37.139 ± 0.164 ^a,b^
C20:3n3	16.541 ± 0.047	21.163 ± 0.049 ^a^	20.906 ± 0.106 ^a^
C20:3n4	9.804 ± 0.028	14.784 ± 0.033 ^a^	15.770 ± 0.069 ^a^
C20:3n6	15.310 ± 0.043	19.079 ± 0.044 ^a^	26.214 ± 0.116 ^a,b^
C22:6	101.980 ± 0.288	122.416 ± 0.281 ^a^	158.623 ± 0.700 ^a,b^
**Heart**	**C group**	**S group**	**M+S group**
L carnitine	0.884 ± 0.003	0.983 ± 0.004 ^a^	1.017 ± 0.004 ^a,b^
TGAs	0.467 ± 0.006	0.651 ± 0.005 ^a^	0.233 ± 0.003 ^a,b^
Glycerol	2.076 ± 0.007	1.952 ± 0.007 ^a^	1.274 ± 0.005 ^a,b^
FFAs	1512.182 ± 3.536	1402.85 ± 6.708 ^a^	1371.662 ± 7.284 ^a,b^
Glucose	0.792 ± 0.001	1.850 ± 0.002 ^a^	0.737 ± 0.003 ^a,b^
Lactate	4.398 ± 0.005	4.870 ± 0.019 ^a^	4.706 ± 0.010 ^a,b^
Inositol	0.467 ± 0.074	0.921 ± 0.003 ^a^	0.738 ± 0.001 ^a,b^
C14:0	43.859 ± 0.010	35.237 ± 0.157 ^a^	28.021 ± 0.148 ^a,b^
C15:0	4.610 ± 0.011	5.741 ± 0.0253 ^a^	4.559 ± 0.0265 ^b^
C16:0	127.17 ± 0.291	120.423 ± 0.538 ^a^	121.225 ± 0.655 ^a^
C16:1	40.896 ± 0.0925	33.153 ± 0.148 ^a^	24.578 ± 0.130 ^a,b^
C17:0	14.158 ± 0.028	10.379 ± 0.045 ^a^	8.072 ± 0.048 ^a,b^
C17:1	6.604 ± 0.013	4.431 ± 0.020 ^a^	3.299 ± 0.017 ^a,b^
C18:0	196.863 ± 0.448	178.384 ± 0.841 ^a^	165.965 ± 0.961 ^a,b^
C18:1t	162.939 ± 0.348	149.855 ± 0.669 ^a^	134.488 ± 0.768 ^a,b^
C18:1c	103.710 ± 0.236	130.540 ± 0.583 ^a^	119.179 ± 0.632 ^a,b^
C18:2c+t	194.688 ± 0.421	180.918 ± 0.807 ^a^	166.481 ± 0.884 ^a,b^
C18:3n3c+t	49.210 ± 0.112	42.275 ± 0.189 ^a^	35.112 ± 0.186 ^a,b^
C20	233.621 ± 0.509	207.710 ± 0.927 ^a^	197.160 ± 1.047 ^a,b^
C20:1	53.837 ± 0.123	36.157 ± 0.161 ^a^	28.730 ± 0.153 ^a,b^
C20:2	51.895 ± 0.119	50.704 ± 0.227	44.225 ± 0.235
C20:3n3	42.273 ± 0.096	33.790 ± 0.160 ^a^	25.986 ± 0.137 ^a,b^
C20:3n4	38.309 ± 0.088	29.691 ± 0.132 ^a^	20.218 ± 0.108 ^a,b^
C20:3n6	33.590 ± 0.077	27.084 ± 0.130 ^a^	14.846 ± 0.078 ^a,b^
C22:6	186.953 ± 0.425	212.379 ± 0.948 ^a^	211.119 ± 1.069 ^a^

**Table 4:** Adrenal glands (μg/g) and serum (pg/mL) noradrenaline and adrenaline concentrations, and serum lipidomics in rats of control (C), sepsis (S) and meldonium + sepsis (M + S) groups. Triglycerides (TGAs), glycerol, total free fatty acid content (FFAs), glucose, lactate and inositol are expressed in mg/g. The number of animals per experimental group: *n* = 8. The data are given as mean ± standard error of the mean. Minimal significant level: *p* < 0.05. Significantly different: ^a^ with respect to the C group; ^b^ with respect to the S group.

Adrenal Glands	C Group	S Group	M + S Group
Noradrenaline	165.320 ± 4.300	139.705 ± 4.032 ^a^	129.545 ± 2.345 ^a^
Adrenaline	720.924 ± 28.162	484.769 ± 14.267 ^a^	510.224 ± 22.258 ^a^
**Serum**	**C group**	**S group**	**M + S group**
Noradrenaline	1764.275 ± 85.679	3116.625 ± 144.720 ^a^	1765.050 ± 98.654 ^a,b^
Adrenaline	1968.475 ± 92.005	4091.450 ± 229.687 ^a^	4159.283 ± 202.225 ^a^
TGAs	1.194 ± 0.004	0.860 ± 0.005 ^a^	0.516 ± 0.003 ^a,b^
Glycerol	1.591 ± 0.005	5.064 ± 0.042 ^a^	0.820 ± 0.001 ^a,b^
FFAs	1976.773 ± 38.599	1428.852 ± 2.615 ^a^	1399.508 ± 1.32 ^a^
Glucose	4.228 ± 0.023	6.473 ± 0.045 ^a^	2.187 ± 0.010 ^a,b^
Lactate	0.224 ± 0.003	0.647 ± 0.002 ^a^	0.705 ± 0.001 ^a^
Inositol	2.116 ± 0.020	5.044 ± 0.017 ^a^	3.654 ± 0.005 ^a,b^

**Table 5:** The study designs. C—control (sham) group of animals (free access to tap water for four weeks and then received a saline injection); S—septic group of animals (free access to tap water for four weeks and then received a faecal intraperitoneal injection); and M + S—meldonium-septic group of animals (free access to tap water with meldonium for four weeks and then received a faecal intraperitoneal injection). The number of animals per group = 8.

Groups	C Group	S Group	M + S Group
Application of meldonium in water	none	none	+
Intraperitoneal application of saline	+	none	none
Intraperitoneal application of LPS	none	+	+

## Data Availability

The data underlying this article will be shared on reasonable request to the corresponding author.

## Supplementary Materials

The following supporting information can be downloaded at: https://www.mdpi.com/article/10.3390/ijms23042395/s1.
