# Supplementary material for: The Effects of a Meldonium Pre-Treatment on the Course of the LPS-Induced Sepsis in Rats
Source: Int J Mol Sci. 2022 Feb 21;23(4):2395. doi: 10.3390/ijms23042395 (PMC8924897; doi:10.3390/ijms23042395)

Principal Components Report

Page/Date/Time 1 01-Dec-21 21:26:02
Database

Descriptive Statistics Section

Variables	Count	Mean	Standard Deviation	Communality
C1	18	64.05431	59.66594	1.000000
C2	18	73.90549	66.80447	1.000000
C3	18	89.0934	77.88059	1.000000

Correlation Section

	Variables		
Variables	C1	C2	C3
C1	1.000000	0.997951	0.995938
C2	0.997951	1.000000	0.996108
C3	0.995938	0.996108	1.000000

Phi=0.996666 Log(Det|R|)=-10.472501 Bartlett Test=158.83 DF=3 Prob=0.000000

Bar Chart of Absolute Correlation Section

	Variables		
Variables	C1	C2	C3
C1		||||||||||||||||||||	||||||||||||||||||||
C2	||||||||||||||||||||		||||||||||||||||||||
C3	||||||||||||||||||||	||||||||||||||||||||	

Phi=0.996666 Log(Det|R|)=-10.472501 Bartlett Test=158.83 DF=3 Prob=0.000000

Eigenvalues

No.	Eigenvalue	Individual Percent	Cumulative Percent	Scree Plot
1	2.993331	99.78	99.78	||||||||||||||||||||
2	0.004623	0.15	99.93	|
3	0.002045	0.07	100.00	|

Eigenvectors

	Factors		
Variables	Factor1	Factor2	Factor3
C1	-0.577458	0.434754	0.691037
C2	-0.577491	0.380798	-0.722147
C3	-0.577102	-0.816077	0.031171

Principal Components Report

Page/Date/Time 2 01-Dec-21 21:26:02
Database

Bar Chart of Absolute Eigenvectors

<table>
<tr><th></th><th>Factors</th><th></th><th></th></tr>
<tr><th>Variables</th><th>Factor1</th><th>Factor2</th><th>Factor3</th></tr>
<tr><td>C1</td><td>||||||||||||</td><td>|||||||||</td><td>||||||||||||||</td></tr>
<tr><td>C2</td><td>||||||||||||</td><td>||||||||</td><td>|||||||||||||||</td></tr>
<tr><td>C3</td><td>||||||||||||</td><td>|||||||||||||||||</td><td>|</td></tr>
</table>

Factor Loadings

	Factors		
**Variables**	**Factor1**	**Factor2**	**Factor3**
C1	-0.999074	0.029561	0.031252
C2	-0.999131	0.025892	-0.032659
C3	-0.998458	-0.055489	0.001410

Bar Chart of Absolute Factor Loadings

<table>
<tr><th></th><th>Factors</th><th></th><th></th></tr>
<tr><th>Variables</th><th>Factor1</th><th>Factor2</th><th>Factor3</th></tr>
<tr><td>C1</td><td>||||||||||||||||||||</td><td>|</td><td>|</td></tr>
<tr><td>C2</td><td>||||||||||||||||||||</td><td>|</td><td>|</td></tr>
<tr><td>C3</td><td>||||||||||||||||||||</td><td>||</td><td>|</td></tr>
</table>

Communalities

	Factors			
**Variables**	**Factor1**	**Factor2**	**Factor3**	**Communality**
C1	0.998149	0.000874	0.000977	1.000000
C2	0.998263	0.000670	0.001067	1.000000
C3	0.996919	0.003079	0.000002	1.000000

Bar Chart of Communalities

<table>
<tr><th></th><th>Factors</th><th></th><th></th><th></th></tr>
<tr><th>Variables</th><th>Factor1</th><th>Factor2</th><th>Factor3</th><th>Communality</th></tr>
<tr><td>C1</td><td>||||||||||||||||||||</td><td>|</td><td>|</td><td>||||||||||||||||||||</td></tr>
<tr><td>C2</td><td>||||||||||||||||||||</td><td>|</td><td>|</td><td>||||||||||||||||||||</td></tr>
<tr><td>C3</td><td>||||||||||||||||||||</td><td>|</td><td>|</td><td>||||||||||||||||||||</td></tr>
</table>

Factor Structure Summary

	Factors	
**Factor1**	**Factor2**	**Factor3**
C2		
C1		
C3		

Residual Section

Row	T2	T2 Prob	Q0	Q1	Q2
1	1.22	0.7833	0.03	0.00	0.00
2	.97	0.8342	2.70	0.00	0.00
3	7.41	0.1330	4.99	0.01	0.01*
4	.84	0.8620	0.66	0.00	0.00
5	.96	0.8382	2.24	0.00	0.00
6	.90	0.8502	2.62	0.00	0.00
7	8.59	0.0967	14.40	0.01	0.01*
8	4.26	0.3259	0.86	0.01	0.00
9	1.32	0.7641	0.44	0.01	0.00
10	6.47	0.1724	8.34	0.01	0.00
11	1.19	0.7889	0.92	0.00	0.00
12	3.62	0.3936	1.83	0.01	0.00
13	.53	0.9242	1.48	0.00	0.00
14	.94	0.8412	1.34	0.00	0.00
15	.96	0.8381	1.96	0.00	0.00
16	1.28	0.7707	2.50	0.00	0.00
17	.69	0.8934	1.99	0.00	0.00
18	8.85	0.0903	1.73	0.03*	0.00

Principal Components Report

Page/Date/Time 4 01-Dec-21 21:26:02
Database

Factor Score

	Factors		
**Row**	**Factor1**	**Factor2**	**Factor3**
1	-0.0894	-0.2148	-1.0803
2	0.9498	0.1635	0.2149
3	-1.2893	0.9377	-2.2061
4	0.4677	-0.6280	-0.4770
5	0.8658	0.4519	0.0425
6	0.9356	0.1136	0.0989
7	-2.1925	0.0245	1.9454
8	-0.5300	-1.6215	1.1617
9	-0.3791	-1.0821	0.0249
10	-1.6677	1.6993	-0.8968
11	0.5526	0.7091	0.6213
12	-0.7790	0.9895	1.4259
13	0.7028	0.0848	0.1717
14	0.6678	0.0234	0.7034
15	0.8085	0.5327	-0.1378
16	0.9129	0.5298	-0.4105
17	0.8159	-0.1295	0.0654
18	-0.7523	-2.5840	-1.2675

Plots Section

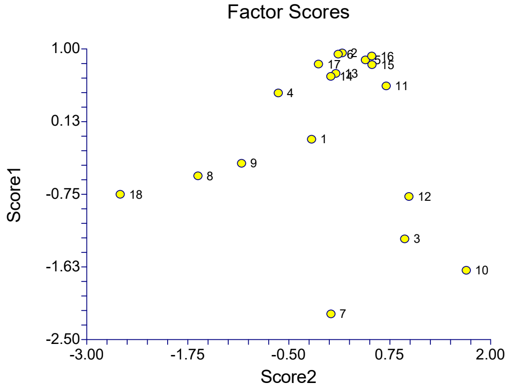

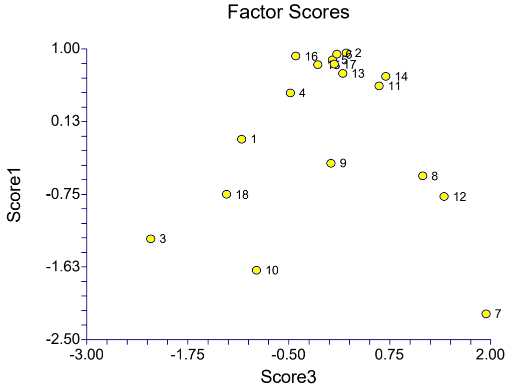

Principal Components Report

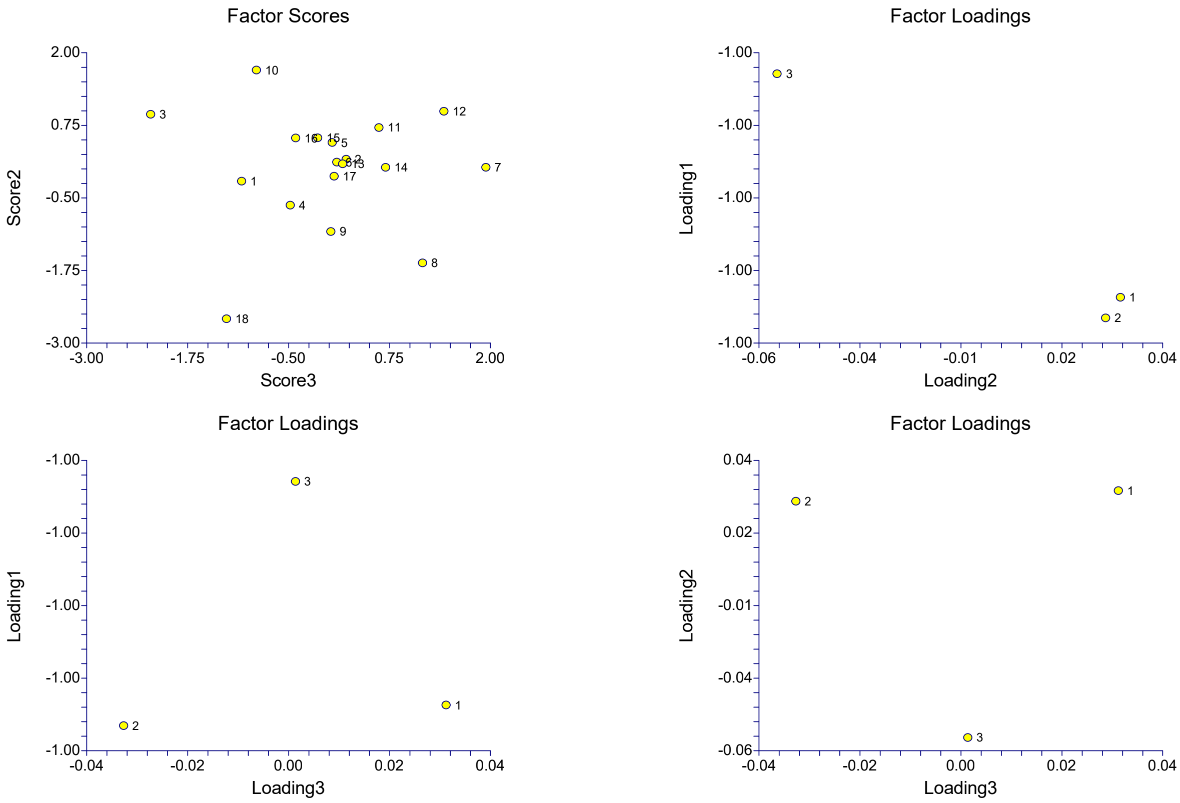

Supplement: Supplementary file 1 [file ijms-23-02395-s001.zip › PCA.zip!/Kidney PCA analysis.pdf]
