# Supplementary material for: The Effects of a Meldonium Pre-Treatment on the Course of the LPS-Induced Sepsis in Rats
Source: Int J Mol Sci. 2022 Feb 21;23(4):2395. doi: 10.3390/ijms23042395 (PMC8924897; doi:10.3390/ijms23042395)

Principal Components Report

Page/Date/Time 1 01-Dec-21 21:10:57
Database D:\ONEDRIVE\LPS LIPIDOMIKA\KLASTERNA JETRA.S0

Descriptive Statistics Section

Variables	Count	Mean	Standard Deviation	Communality
jetra_c	18	123.1307	119.3745	1.000000
jetra_s	18	138.1656	124.9316	1.000000
jetra_m_s	18	159.904	151.8847	1.000000

Correlation Section

	Variables		
Variables	jetra_c	jetra_s	jetra_m_s
jetra_c	1.000000	0.985982	0.973163
jetra_s	0.985982	1.000000	0.991668
jetra_m_s	0.973163	0.991668	1.000000

Phi=0.983635 Log(Det|R|)=-7.726903 Bartlett Test=117.19 DF=3 Prob=0.000000

Bar Chart of Absolute Correlation Section

	Variables		
Variables	jetra_c	jetra_s	jetra_m_s
jetra_c		||||||||||||||||||||	||||||||||||||||||||
jetra_s	||||||||||||||||||||		||||||||||||||||||||
jetra_m_s	||||||||||||||||||||	||||||||||||||||||||	

Phi=0.983635 Log(Det|R|)=-7.726903 Bartlett Test=117.19 DF=3 Prob=0.000000

Eigenvalues

No.	Eigenvalue	Individual Percent	Cumulative Percent	Scree Plot
1	2.967229	98.91	98.91	||||||||||||||||||||
2	0.027337	0.91	99.82		
3	0.005434	0.18	100.00		

Eigenvectors

	Factors		
Variables	Factor1	Factor2	Factor3
jetra_c	-0.575770	0.761426	0.297857
jetra_s	-0.579385	-0.122926	-0.805731
jetra_m_s	-0.576890	-0.636490	0.511936

Principal Components Report

Page/Date/Time 2 01-Dec-21 21:10:57
Database D:\ONEDRIVE\LPS LIPIDOMIKA\KLASTERNA JETRA.S0

Bar Chart of Absolute Eigenvectors

<table>
<tr><th></th><th>Factors</th><th></th><th></th></tr>
<tr><th>Variables</th><th>Factor1</th><th>Factor2</th><th>Factor3</th></tr>
<tr><td>jetra_c</td><td>||||||||||||</td><td>||||||||||||||||</td><td>||||||</td></tr>
<tr><td>jetra_s</td><td>||||||||||||</td><td>|||</td><td>|||||||||||||||||</td></tr>
<tr><td>jetra_m_s</td><td>||||||||||||</td><td>|||||||||||||</td><td>|||||||||||</td></tr>
</table>

Factor Loadings

	Factors		
**Variables**	**Factor1**	**Factor2**	**Factor3**
jetra_c	-0.991801	0.125893	0.021958
jetra_s	-0.998027	-0.020324	-0.059397
jetra_m_s	-0.993731	-0.105236	0.037739

Bar Chart of Absolute Factor Loadings

<table>
<tr><th></th><th>Factors</th><th></th><th></th></tr>
<tr><th>Variables</th><th>Factor1</th><th>Factor2</th><th>Factor3</th></tr>
<tr><td>jetra_c</td><td>||||||||||||||||||||</td><td>|||</td><td>|</td></tr>
<tr><td>jetra_s</td><td>||||||||||||||||||||</td><td>|</td><td>||</td></tr>
<tr><td>jetra_m_s</td><td>||||||||||||||||||||</td><td>|||</td><td>|</td></tr>
</table>

Communalities

	Factors			
**Variables**	**Factor1**	**Factor2**	**Factor3**	**Communality**
jetra_c	0.983669	0.015849	0.000482	1.000000
jetra_s	0.996059	0.000413	0.003528	1.000000
jetra_m_s	0.987501	0.011075	0.001424	1.000000

Bar Chart of Communalities

<table>
<tr><th></th><th>Factors</th><th></th><th></th><th></th></tr>
<tr><th>Variables</th><th>Factor1</th><th>Factor2</th><th>Factor3</th><th>Communality</th></tr>
<tr><td>jetra_c</td><td>||||||||||||||||||||</td><td>|</td><td>|</td><td>||||||||||||||||||||</td></tr>
<tr><td>jetra_s</td><td>||||||||||||||||||||</td><td>|</td><td>|</td><td>||||||||||||||||||||</td></tr>
<tr><td>jetra_m_s</td><td>||||||||||||||||||||</td><td>|</td><td>|</td><td>||||||||||||||||||||</td></tr>
</table>

Principal Components Report

Page/Date/Time 3 01-Dec-21 21:10:57
Database D:\ONEDRIVE\LPS LIPIDOMIKA\KLASTERNA JETRA.S0

Factor Structure Summary

	Factors	
**Factor1**	**Factor2**	**Factor3**
jetra_s		
jetra_m_s		
jetra_c		

Residual Section

Row	T2	T2 Prob	Q0	Q1	Q2
1	.11	0.9918	0.02	0.00	0.00
2	.80	0.8702	2.29	0.00	0.00
3	7.90	0.1162	2.43	0.15*	0.01
4	.81	0.8690	1.12	0.01	0.00
5	.77	0.8773	2.25	0.00	0.00
6	1.16	0.7952	2.66	0.00	0.00
7	8.18	0.1080	14.10	0.02	0.02
8	.43	0.9437	1.10	0.00	0.00
9	3.14	0.4534	0.72	0.03	0.01
10	11.12	0.0507	7.82	0.08	0.04*
11	.74	0.8821	1.08	0.00	0.00
12	1.36	0.7556	2.34	0.01	0.00
13	.77	0.8764	1.53	0.00	0.00
14	.80	0.8696	1.03	0.01	0.00
15	.72	0.8862	2.09	0.00	0.00
16	.87	0.8557	2.51	0.00	0.00
17	.75	0.8809	2.16	0.00	0.00
18	10.57	0.0582	3.76	0.24*	0.00

Principal Components Report

Page/Date/Time 4 01-Dec-21 21:10:57
Database D:\ONEDRIVE\LPS LIPIDOMIKA\KLASTERNA JETRA.S0

Factor Score

	Factors		
**Row**	**Factor1**	**Factor2**	**Factor3**
1	-0.0870	0.2842	0.1474
2	0.8776	-0.0561	0.1652
3	-0.8751	2.2969	1.3647
4	0.6111	0.5667	0.3346
5	0.8711	0.0842	0.0132
6	0.9463	0.2335	0.4621
7	-2.1782	0.1745	1.8448
8	-0.6085	-0.2362	0.0009
9	-0.4829	-0.7868	-1.5124
10	-1.6156	1.1907	-2.6637
11	0.6006	0.3651	0.4987
12	-0.8865	-0.4006	-0.6407
13	0.7165	-0.1833	-0.4726
14	0.5881	-0.3595	-0.5734
15	0.8395	-0.1332	0.0067
16	0.9194	-0.1581	0.0278
17	0.8530	0.0562	0.1323
18	-1.0895	-2.9380	0.8644

Plots Section

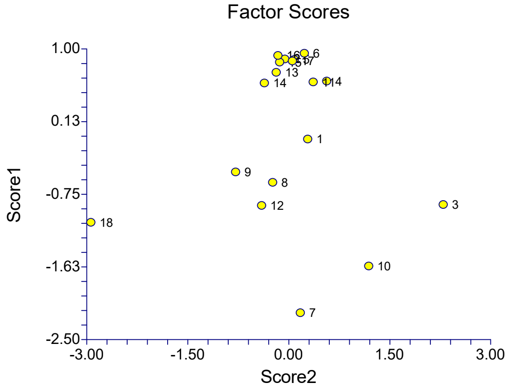

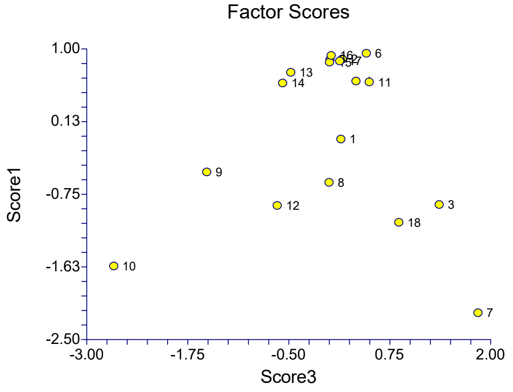

Principal Components Report

Page/Date/Time 5 01-Dec-21 21:10:57
Database D:\ONEDRIVE\LPS LIPIDOMIKA\KLASTERNA JETRA.S0

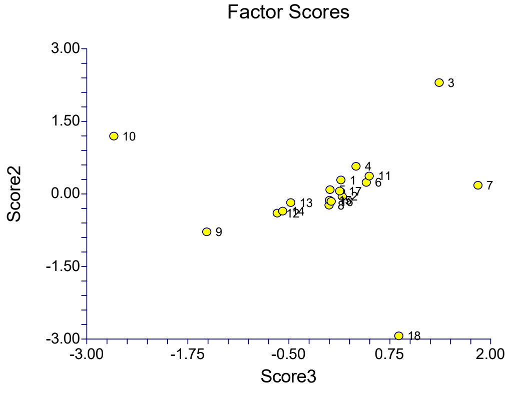

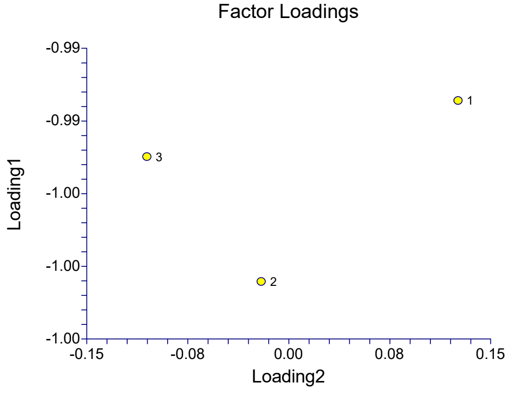

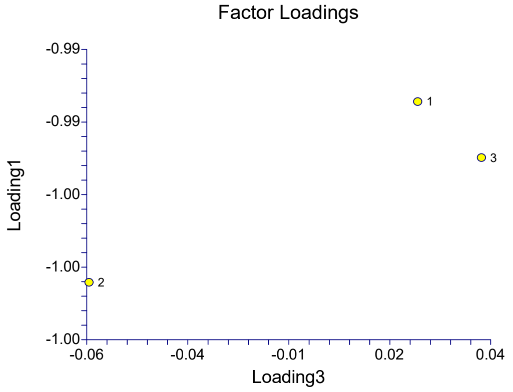

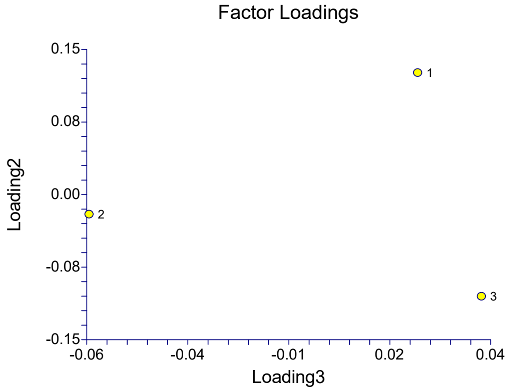

Supplement: Supplementary file 1 [file ijms-23-02395-s001.zip › PCA.zip!/Liver PCA analysis.pdf]
