# Supplementary material for: The Effects of a Meldonium Pre-Treatment on the Course of the LPS-Induced Sepsis in Rats
Source: Int J Mol Sci. 2022 Feb 21;23(4):2395. doi: 10.3390/ijms23042395 (PMC8924897; doi:10.3390/ijms23042395)

Principal Components Report

Page/Date/Time	1	01-Dec-21 21:37:55
Database		

Descriptive Statistics Section

Variables	Count	Mean	Standard Deviation	Communality
C1	18	86.23236	72.95625	1.000000
C2	18	83.49167	75.21184	1.000000
C3	18	76.20348	73.70448	1.000000

Correlation Section

	Variables		
Variables	C1	C2	C3
C1	1.000000	0.988327	0.987117
C2	0.988327	1.000000	0.998203
C3	0.987117	0.998203	1.000000

Phi=0.991228 Log(Det|R|)=-9.396265 Bartlett Test=142.51 DF=3 Prob=0.000000

Bar Chart of Absolute Correlation Section

<table>
<tr><th></th><th>Variables</th><th></th><th></th></tr>
<tr><th>Variables</th><th>C1</th><th>C2</th><th>C3</th></tr>
<tr><td>C1</td><td></td><td>||||||||||||||||||||</td><td>||||||||||||||||||||</td></tr>
<tr><td>C2</td><td>||||||||||||||||||||</td><td></td><td>||||||||||||||||||||</td></tr>
<tr><td>C3</td><td>||||||||||||||||||||</td><td>||||||||||||||||||||</td><td></td></tr>
</table>

Phi=0.991228 Log(Det|R|)=-9.396265 Bartlett Test=142.51 DF=3 Prob=0.000000

Eigenvalues

<table>
<tr><th>No.</th><th>Eigenvalue</th><th>Individual Percent</th><th>Cumulative Percent</th><th>Scree Plot</th></tr>
<tr><td>1</td><td>2.982440</td><td>99.41</td><td>99.41</td><td>||||||||||||||||||||</td></tr>
<tr><td>2</td><td>0.015798</td><td>0.53</td><td>99.94</td><td>|</td></tr>
<tr><td>3</td><td>0.001762</td><td>0.06</td><td>100.00</td><td>|</td></tr>
</table>

Eigenvectors

	Factors		
Variables	Factor1	Factor2	Factor3
C1	-0.575990	-0.816434	0.040878
C2	-0.578146	0.371506	-0.726450
C3	-0.577912	0.442062	0.686002

Principal Components Report

Page/Date/Time 2 01-Dec-21 21:37:55
Database

Bar Chart of Absolute Eigenvectors

<table>
<tr><th></th><th>Factors</th><th></th><th></th></tr>
<tr><th>Variables</th><th>Factor1</th><th>Factor2</th><th>Factor3</th></tr>
<tr><td>C1</td><td>||||||||||||</td><td>|||||||||||||||||</td><td>|</td></tr>
<tr><td>C2</td><td>||||||||||||</td><td>||||||||</td><td>|||||||||||||||</td></tr>
<tr><td>C3</td><td>||||||||||||</td><td>|||||||||</td><td>||||||||||||||</td></tr>
</table>

Factor Loadings

	Factors		
**Variables**	**Factor1**	**Factor2**	**Factor3**
C1	-0.994719	-0.102618	0.001716
C2	-0.998444	0.046695	-0.030496
C3	-0.998040	0.055563	0.028798

Bar Chart of Absolute Factor Loadings

<table>
<tr><th></th><th>Factors</th><th></th><th></th></tr>
<tr><th>Variables</th><th>Factor1</th><th>Factor2</th><th>Factor3</th></tr>
<tr><td>C1</td><td>||||||||||||||||||||</td><td>|||</td><td>|</td></tr>
<tr><td>C2</td><td>||||||||||||||||||||</td><td>|</td><td>|</td></tr>
<tr><td>C3</td><td>||||||||||||||||||||</td><td>||</td><td>|</td></tr>
</table>

Communalities

	Factors			
**Variables**	**Factor1**	**Factor2**	**Factor3**	**Communality**
C1	0.989467	0.010531	0.000003	1.000000
C2	0.996890	0.002180	0.000930	1.000000
C3	0.996083	0.003087	0.000829	1.000000

Bar Chart of Communalities

<table>
<tr><th></th><th>Factors</th><th></th><th></th><th></th></tr>
<tr><th>Variables</th><th>Factor1</th><th>Factor2</th><th>Factor3</th><th>Communality</th></tr>
<tr><td>C1</td><td>||||||||||||||||||||</td><td>|</td><td>|</td><td>||||||||||||||||||||</td></tr>
<tr><td>C2</td><td>||||||||||||||||||||</td><td>|</td><td>|</td><td>||||||||||||||||||||</td></tr>
<tr><td>C3</td><td>||||||||||||||||||||</td><td>|</td><td>|</td><td>||||||||||||||||||||</td></tr>
</table>

Principal Components Report

Page/Date/Time 3 01-Dec-21 21:37:55
Database

Factor Structure Summary

	Factors	
**Factor1**	**Factor2**	**Factor3**
C2		
C3		
C1		

Residual Section

Row	T2	T2 Prob	Q0	Q1	Q2
1	.59	0.9119	1.18	0.00	0.00
2	2.75	0.5079	3.25	0.01	0.00
3	6.45	0.1737	0.96	0.01	0.01*
4	.83	0.8647	1.32	0.00	0.00
5	2.11	0.6127	2.81	0.01	0.00
6	2.36	0.5698	3.30	0.01	0.00
7	2.95	0.4790	6.26	0.01	0.00
8	1.41	0.7443	2.47	0.00	0.00
9	6.86	0.1548	0.79	0.09*	0.00
10	3.25	0.4391	5.00	0.01	0.00
11	.39	0.9508	0.87	0.00	0.00
12	5.49	0.2278	8.97	0.04	0.00
13	1.88	0.6549	1.01	0.02	0.00
14	.26	0.9718	0.60	0.00	0.00
15	1.09	0.8095	1.23	0.00	0.00
16	1.23	0.7812	1.52	0.01	0.00
17	3.92	0.3600	1.74	0.01	0.01
18	7.18	0.1417	7.71	0.07*	0.00

Factor Score

	Factors		
**Row**	**Factor1**	**Factor2**	**Factor3**
1	0.6273	-0.4228	-0.1459
2	1.0427	0.8120	1.0035
3	-0.5650	0.0503	2.4746
4	0.6657	-0.4053	-0.4692
5	0.9689	0.5186	0.9493
6	1.0515	0.5928	0.9502
7	-1.4483	-0.7287	0.5700
8	-0.9104	-0.0727	0.7609
9	-0.4844	2.3435	-1.0634
10	-1.2936	-0.6292	-1.0859
11	0.5393	-0.2843	-0.1214
12	-1.7302	-1.5788	0.0712
13	0.5743	-1.2413	-0.0672
14	0.4481	0.2427	-0.0045
15	0.6413	-0.3570	-0.7453
16	0.7127	-0.5190	-0.6741
17	0.7614	-0.3791	-1.7881
18	-1.6013	2.0583	-0.6147

Plots Section

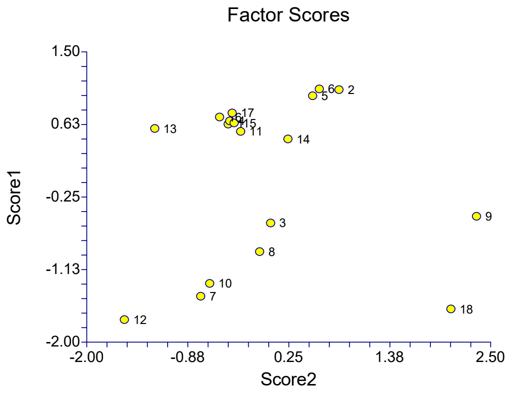

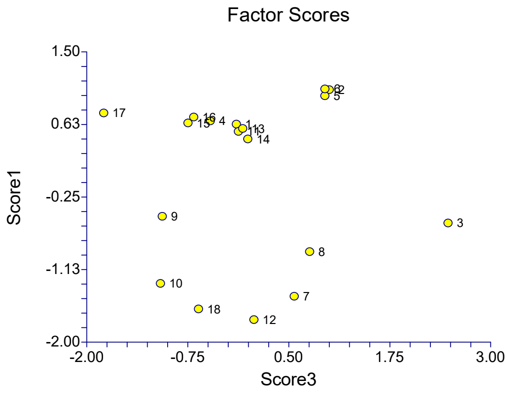

Principal Components Report

Page/Date/Time 5 01-Dec-21 21:37:55
Database

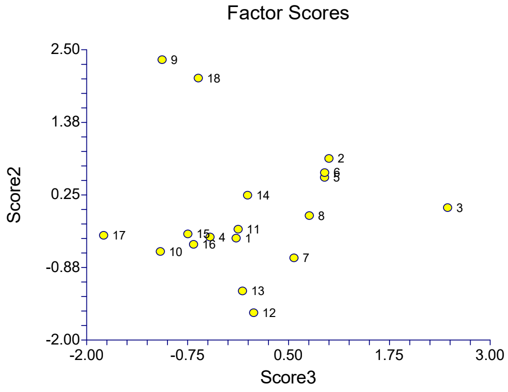

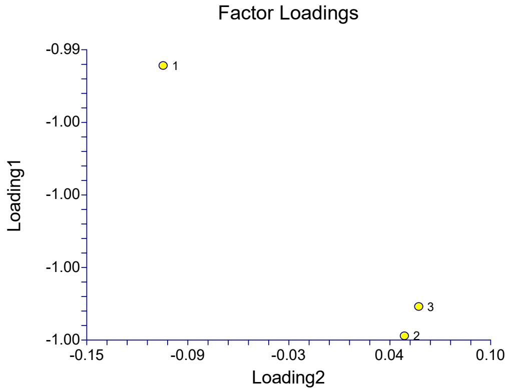

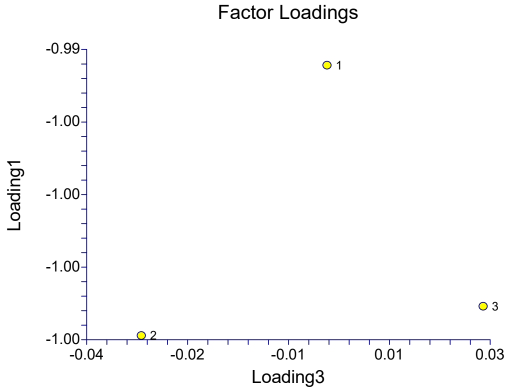

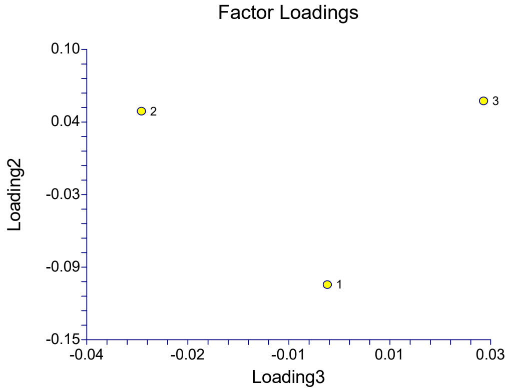

Supplement: Supplementary file 1 [file ijms-23-02395-s001.zip › PCA.zip!/Heart PCA analysis.pdf]
